# Regulation of synaptic connectivity in schizophrenia spectrum by mutual neuron-microglia interaction

**DOI:** 10.1038/s42003-023-04852-9

**Published:** 2023-04-29

**Authors:** Ricarda Breitmeyer, Sabrina Vogel, Johanna Heider, Sophia-Marie Hartmann, Richard Wüst, Anna-Lena Keller, Anna Binner, Julia C. Fitzgerald, Andreas J. Fallgatter, Hansjürgen Volkmer

**Affiliations:** 1 Molecular Neurobiology, Department of Pharma and Biotech, NMI Natural and Medical Sciences Institute at the University of Tübingen, 72770 Reutlingen, Germany; 2 Department of Psychiatry, Tübingen Center for Mental Health (TüCMH), University of Tübingen, Osianderstrasse 24, 72076 Tübingen, Germany; 3 Tumor Biology, Department of Pharma and Biotech, NMI Natural and Medical Sciences Institute at the University of Tübingen, 72770 Reutlingen, Germany; 4 Hertie Institute for Clinical Brain Research, Otfried-Mueller-Strasse 27, 72076 Tübingen, Germany

**Keywords:** Schizophrenia, Induced pluripotent stem cells

## Abstract

The examination of post-mortem brain tissue suggests synaptic loss as a central pathological hallmark of schizophrenia spectrum (SCZ), which is potentially related to activated microglia and increased inflammation. Induced pluripotent stem cells serve as a source for neurons and microglia-like cells to address neuron-microglia interactions. Here, we present a co-culture model of neurons and microglia, both of human origin, to show increased susceptibility of neurons to microglia-like cells derived from SCZ patients. Analysis of IBA-1 expression, NFκB signaling, transcription of inflammasome-related genes, and caspase-1 activation shows that enhanced, intrinsic inflammasome activation in patient-derived microglia exacerbates neuronal deficits such as synaptic loss in SCZ. Anti-inflammatory pretreatment of microglia with minocycline specifically rescued aberrant synapse loss in SCZ and reduced microglial activation. These findings open up possibilities for further research in larger cohorts, focused clinical work and longitudinal studies that could facilitate earlier therapeutic intervention.

## Abstract (web summary)

A patient iPSC co-culture model is developed to study neuron-microglia interactions during neuroinflammation to understand how microglial activation contributes to schizophrenia-related synaptic and neuronal pathologies

## Introduction

Schizophrenia spectrum (SCZ) is a complex and highly heterogeneous mental disorder characterized by severe disabling social and clinical impairments. The broad range of individual psychotic symptoms includes hallucinations, delusions, apathy and withdrawal, as well as cognitive deficits. Diverse symptoms in combination with a huge variability between individual patients complicates the understanding of underlying biological causes and hampers the development of novel therapeutics. So far, antipsychotic drug application aims to reduce symptom severity and improve quality of life, but there is currently no cure available. Genetic predispositions, prenatal stress or social and environmental factors have been suggested to contribute to disease pathology. A majority of research focuses on altered neurobiological function, such as deregulated neurotransmitter release and aberrant neuronal activity. More recently, epidemiological studies and genome-wide association studies suggested a link between SCZ and prenatal infection, systemic inflammation and immune system dysfunction^1–5^.

Microglia that comprise one population of immune cells of the central nervous system, emerge as key regulators of early neurodevelopment and synaptic plasticity, while governing neuroimmunological responses. In SCZ, aberrant neuroinflammation mediated by reactive microglia may account for synaptic and neuronal pathologies. PET imaging revealed elevated microglial activation in SCZ patients, while peripheral cytokine levels were increased^6–8^. Likewise, post-mortem tissue analysis and PET imaging of patients repeatedly showed decreased cortical volume and reduced synaptic density^9–12^. These findings strengthened the hypothesis that an increased inflammatory state of microglia is responsible for the observed loss of neuronal connectivity in SCZ. So far, it is not understood how microglial activation in SCZ contributes to the underlying pathology.

The prevailing view of SCZ etiology has relied on mainly peripheral markers, imaging and postmortem studies in patient fibroblasts. Recent advances in human induced pluripotent stem cell (iPSC) models provide a valuable tool for studying disease-relevant and patient-specific mechanisms of SCZ in neurons^13–15^. Examination of the interaction of neurons and microglia in iPSC-derived models remained a challenge in the field, but a complete human model comprising both cell types is greatly needed for SCZ research. So far, microglia-like cells were either generated from blood-derived monocytes and exposed to acellular synaptosome preparations, or human interneurons were co-cultivated with murine microglial cell lines^16,17^.

This prompted us to differentiate neurons and microglial cells from iPSCs of the same donor with its genetic background to setup co-culture models that allow the study of neuron-microglia interactions for the understanding of inflammatory processes in SCZ. Over the last years, several protocols were published for differentiating microglia-like cells from iPSC, yielding functional cells with high similarity compared to primary human microglia cells^18–20^. However, most protocols are time consuming. The protocol presented here represents an accelerated differentiation procedure with precisely timed addition of crucial cytokines and growth factors, especially by high-dose application of IL-34, TGFβ1 and GM-CSF^21^.

Here, we made use of a collection of previously described iPS cell lines from patients with SCZ to establish a fast microglia differentiation protocol^22^. We show that SCZ microglia display an elevated activation state as compared to healthy controls that is linked to increased TNFα secretion and NFκB signaling as well as to enhanced inflammasome activity. Likewise, microglia-like cells were combined with NGN2-induced neurons for an iPSC-derived co-culture that allows for the analysis of neuronal and microglial phenotypes in SCZ via direct cell-cell interactions. When co-cultured with SCZ neurons, SCZ microglia exacerbate the intrinsic deficit of SCZ neurons to form synapses, while SCZ neurons enhance microglial activation. Importantly, anti-inflammatory pretreatment of microglial cells specifically rescues SCZ-associated synaptic loss while having no impact on healthy controls.

## Results

### Differentiation of microglial cells from iPSC

**Fig. 1:**
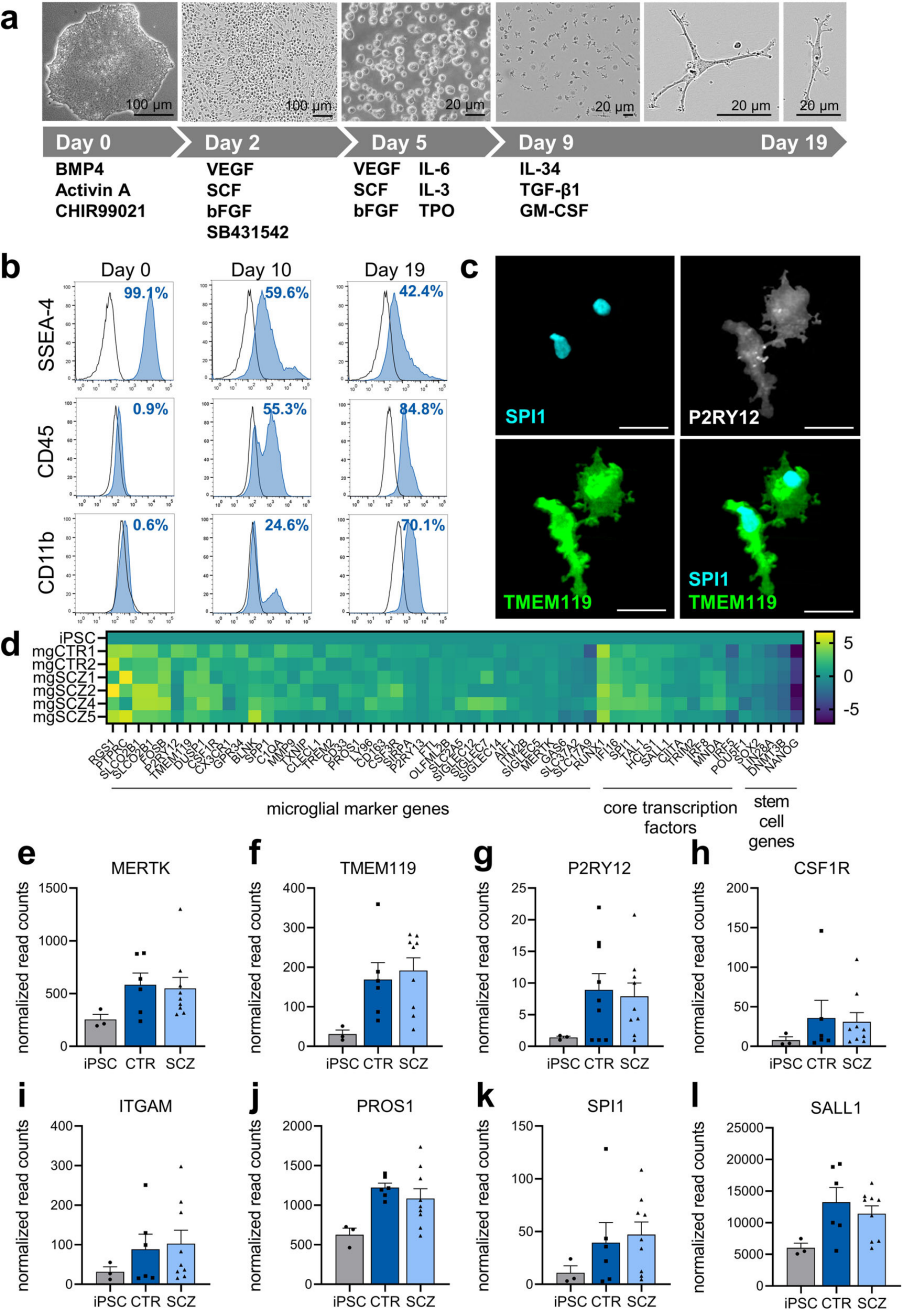
Differentiation of iPSC gives rise to microglia-like cells. **a** Schematic diagram depicting all steps of myeloid differentiation and microglial maturation from iPSC. **b** Exemplary, temporal expression of stem cell marker SSEA-4, myeloid marker CD45 and microglia marker CD11b after onset of differentiation as measured by FACS analysis. **c** Representative immunocytochemistry (63x) of day 19 microglia-like cells shows expression of microglia markers TMEM119 and P2RY12, as well as expression of the transcription factor SPI1. Scale bar 20 µm. **d** Heatmap displaying log2 transformed expression in microglia derived from individual iPSC clones in comparison to naïve iPSC as determined by RNA sequencing. Expression of key marker genes and core transcription factors are upregulated in microglia, while stem cell genes become downregulated. **e**–**l** Normalized expression of microglia signature genes in CTR- and SCZ-microglia was determined by RNA sequencing. Microglial RNA was extracted from untreated, day 19 microglia derived from three independent differentiations for two control and four patient-derived lines. RNA was extracted from iPS cells in different passages. No differences in microglia marker gene expression were detectable indicating that the differentiation protocol is equally efficient for control and patient-derived cells and the cells’ capacity to differentiate is not affected in SCZ. Normalized read counts are represented as mean ± SEM, (e-I: iPSC *n* = 3, CTR *n* = 6, SCZ *n* = 9).

iPS cell lines from two healthy volunteers and four cell lines from four patients with SCZ were used in this study as indicated in Table 1^22,23^. Patients were diagnosed within schizophrenia spectrum (SCZ) according to DSM-IV. Two patients were diagnosed with schizophrenia (paranoid and residual type), while the other two patients were diagnosed with schizoaffective disorder (for detailed information on DSM-diagnosis, symptoms and medication see Supplementary Table 1). Microglia were generated from iPSC by high-dose application of IL-34, TGFβ1 and GM-CSF to optimize specification of microglia (Fig. 1a). Microglia-like cells showed ramified processes indicative of a resting state morphology. Flow cytometry analysis of differentiating cells over 19 days revealed a dynamic downregulation of the stem cell marker SSEA-4, while hematopoietic and microglial markers CD45 and CD11b, respectively, became upregulated (Fig. 1b). Immunocytochemical analysis of microglial marker proteins including the transcription factor SPI1 as well as P2RY12 and TMEM119 confirmed a specific microglial phenotype (Fig. 1c), while immune cell and microglia-specific marker proteins SPI1, CX3CR1 or IBA1 were undetectable in the respective iPSC lines (Supplementary Fig. 1). A pH-sensitive uptake assay with *Escherichia coli*-derived bioparticles revealed phagocytic functions of differentiated microglia (Supplementary Fig. 2). For a more comprehensive analysis of microglia-like cells, we applied RNA sequencing for the comparison of iPSC with microglia-like cells (Fig. 1d). The results indicated downregulation of stem cell genes, while key microglial marker and specific transcription factor genes became upregulated. In detail, RNA sequencing identified upregulation of genes involved in chemokine or cytokine signaling, antigen processing and presentation as well as toll-like receptor signaling in differentiated microglia as compared to naïve iPS cells (Supplementary Fig. 3). Likewise, LPS stimulation increased pro-inflammatory gene expression of NFΚB1, IL-1β, TNFα as well as immune response genes such as CIITA and HLA-DBR1 (Supplementary Fig. 4). iPSC derived from healthy volunteers and patients diagnosed with SCZ displayed no differences in differentiation capacity, microglial cell yield (Supplementary Fig. 5), or differential expression of microglia key genes (Fig. 1e–l). In conclusion, the newly developed differentiation protocol proved to be suitable for the generation of microglia-like cells.

**Table 1:** Cell lines used in this study.

iPS cell lines used in this study	Cell line unique identifier (hpscreg.eu)	Reference
CTR1	NMIi001-A	Stock et al., 2020^22^
CTR2	NMIi010-A	Keller et al., 2021^23^
SCZ1	NMIi002-A	Stock et al., 2020^22^
SCZ2	NMIi004-A	Stock et al., 2020^22^
SCZ4	NMIi005-A	Stock et al., 2020^22^
SCZ5	NMIi006-A	Stock et al., 2020^22^

### Elevation of immune-related transcripts in SCZ microglial cells

**Fig. 2:**
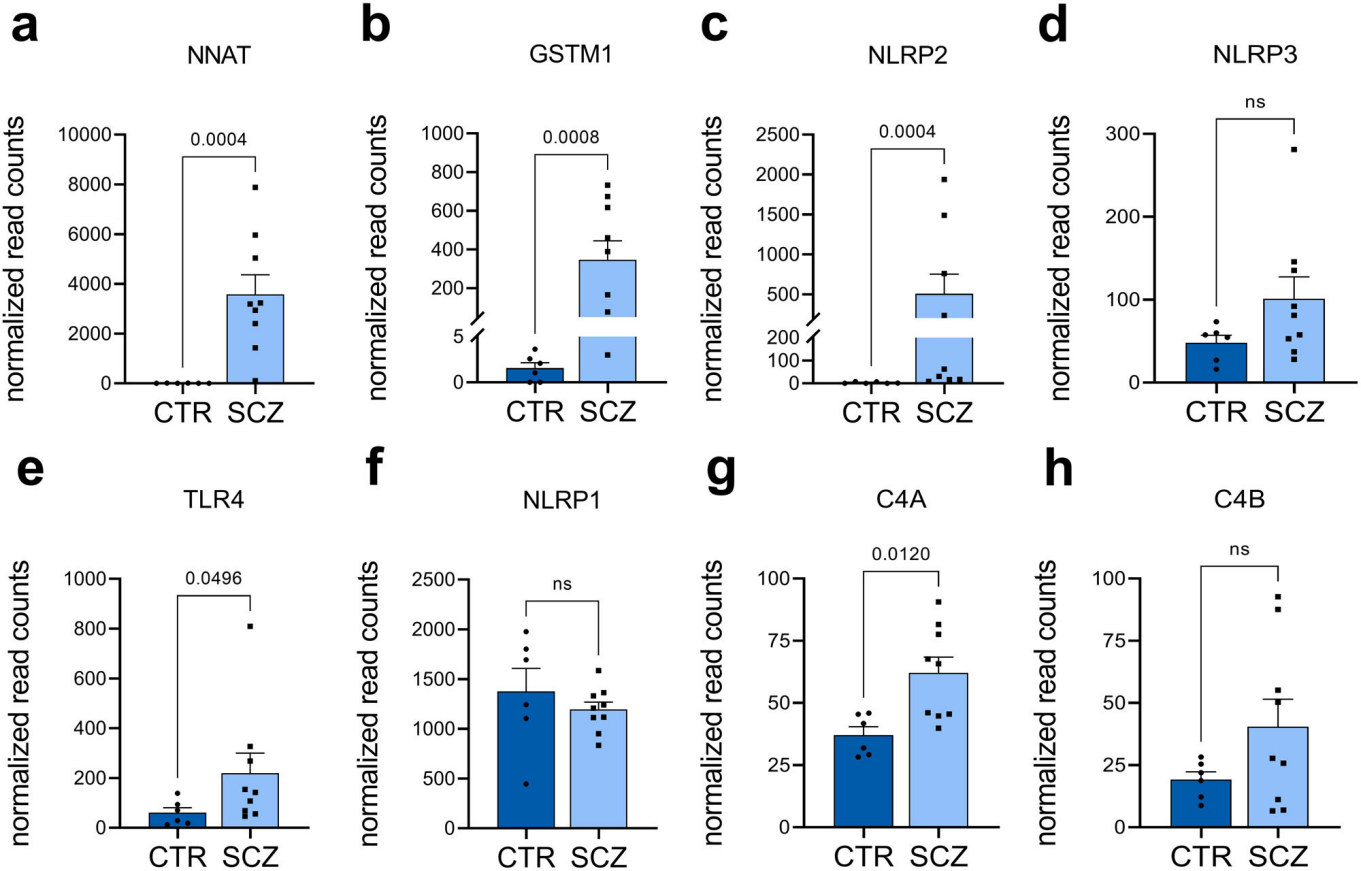
Differential expression of inflammation- and complement-related genes in untreated CTR and SCZ microglia-like cells by RNA sequencing. RNA was extracted from untreated, day 19 microglia derived from three independent differentiations for two control and four patient-derived lines. Unpaired, two-tailed Mann–Whitney U test was employed for pairwise comparisons (a-h: CTR n = 6, SCZ n = 9). Data are represented as mean ± SEM, ns=not significant. **a**, **b** RNA sequencing revealed a significant upregulation of the genes NNAT (*p* = 0.0004) and GSTM1 (*p* = 0.0008) in SCZ microglia compared to CTR microglia. **c**, **d** Quantification of NLRP2 and NLRP3 gene expression, which are involved in the activation of the inflammasome or caspase-1 activity. RNA Sequencing revealed a significant upregulation of NLRP2 in SCZ microglia (*p* = 0.0004). Similarly, NLRP3 expression is slightly increased (*p* = 0.181). **e** TLR4 gene expression is significantly upregulated (*p* = 0.0496) in SCZ microglia. **f** NLRP1 expression is not significantly (*p* = 0.388) altered in SCZ microglia, in contrast to its family members NLRP2 and NLRP3. **g**, **h** Expression of complement and schizophrenia-associated risk gene C4A is significantly increased (*p* = 0.012) in SCZ-microglia, while C4B expression is slightly but not significantly increased (*p* = 0.388).

RNA sequencing was applied to identify deregulated transcripts in SCZ. Among the upregulated genes, we found neuronatin (NNAT), glutathion S-transferase M1 (GSTM1), NLRP2, NLRP3, and TLR4 (Fig. 2a–e, Supplementary Table 2). NLRP2 and 3 as well as TLR4 are linked to NFκB signaling and inflammasome functioning^24^. In contrast, NLRP1 remained equally expressed in healthy control and SCZ samples (Fig. 2f). GSTM1 and NNAT were previously suggested to be involved in inflammation^25,26^. We also observed an increased mRNA expression of the SCZ-risk gene complement factor C4A (Fig. 2g), a further regulator of the innate immune response, which is in agreement with mRNA quantifications performed on patient samples^27^. C4B expression was upregulated in SCZ microglia, although statistical significance was not achieved (Fig. 2h). In summary, microglia-like cells derived from patients with SCZ showed increased expression of genes involved in inflammation.

### Activation of iPSC-derived microglial cells in SCZ

**Fig. 3:**
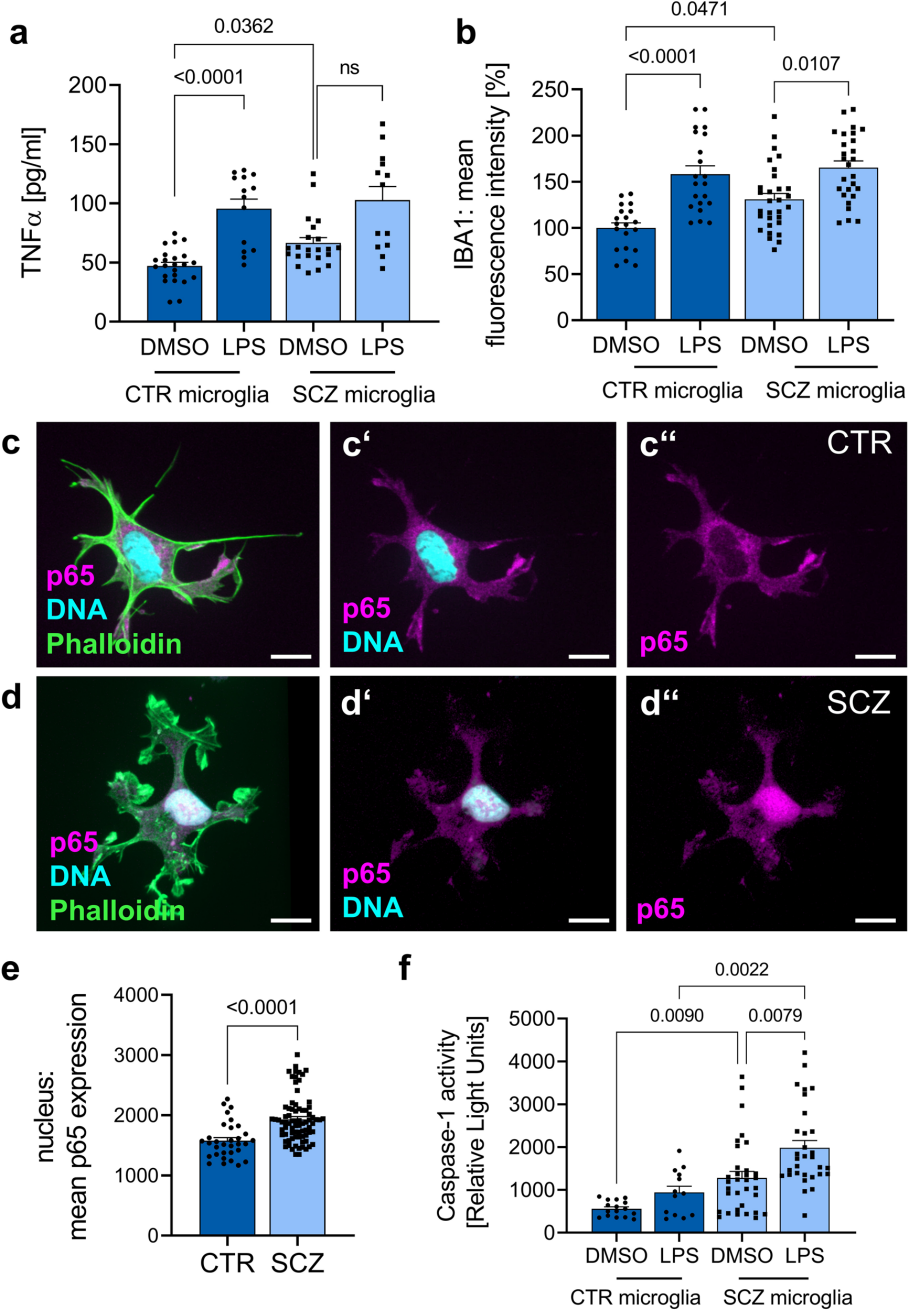
SCZ microglia show a proinflammatory phenotype in combination with significant inflammasome activation. **a** Quantification of TNFα release after LPS treatment (100 ng/ml) for 24 h in comparison to vehicle-treated microglia by a sandwich enzyme-linked immunosorbent assay (ELISA). Kruskal-Wallis test with Dunn’s post hoc test, H(3) = 30.2 (mgCTR DMSO vs. mgCTR LPS *p* < 0.0001, mgCTR DMSO vs. mgSCZ DMSO *p* = 0.0362, mgSCZ DMSO vs. mgSCZ LPS ns). Data represent three independent experiments (CTR microglia DMSO *n* = 23, CTR microglia LPS *n* = 14, SCZ microglia DMSO *n* = 22, SCZ microglia LPS *n* = 13), ns: not significant and mg=microglia. **b** Quantitative analysis of mean IBA1 fluorescence intensity of CTR and SCZ microglia treated with LPS (100 ng/ml) for 24 h as an indirect measurement for microglial activation. Kruskal–Wallis test with Dunn’s post hoc test, H(3) = 34.6 (mgCTR DMSO vs. mgCTR LPS *p* < 0.0001, mgCTR DMSO vs. mgSCZ DMSO *p* = 0.0471, mgSCZ DMSO vs. mgSCZ LPS *p* = 0.0107). Data are represented from three independent experiments (CTR microglia DMSO *n* = 20, CTR microglia LPS *n* = 21, SCZ microglia DMSO *n* = 30, SCZ microglia LPS *n* = 27), ns: not significant. **c**, **d** Representative immunocytochemical stainings of day 19 CTR and SCZ microglia taken at 63x magnification using confocal microscopy. Microglia were stained for Phalloidin, the transcription factor p65 as a subunit of the nuclear factor NF-kappa-B (NFκB) and Hoechst for nucleus visualization. Scale bars 15 µm. **e** p65 expression within the nucleus was quantified and revealed an increased p65 expression in unstimulated SCZ microglia compared to control cells. Unpaired, two-tailed Mann–Whitney U test with a *p*-value of <0.0001. Data are represented from three independent experiments (CTR *n* = 32, SCZ *n* = 78). **f** Caspase-1 activity was further quantified using a bioluminescent assay as a direct readout for inflammasome formation and activation. Cells were treated with 100 ng/ml LPS for 3 h as positive control for inflammasome activation. Kruskal–Wallis test with Dunn’s post hoc test, H(3) = 36.56 (mgCTR DMSO vs. mgCTR LPS *p* = 0.0090, mgCTR LPS vs. mgSCZ LPS *p* = 0.0022, mgSCZ DMSO vs. mgSCZ LPS *p* = 0.0079), indicating increased inflammasome activation in SCZ microglia. Data are represented from three independent experiments (CTR microglia DMSO *n* = 16, CTR microglia LPS *n* = 13, SCZ microglia DMSO *n* = 33, SCZ microglia LPS *n* = 31).

Next, we examined the activated state of microglial cells more closely to link the transcriptomic profiles to microglial functionalities. In support of an enhanced inflammatory state of SCZ microglia, we observed increased TNFα levels in the supernatant of SCZ microglial cells as compared to healthy control microglia (Fig. 3a). In parallel, quantitative imaging revealed upregulated IBA1 expression (Fig. 3b). Increased TNFα levels might induce NFκB signaling after binding to the TNFα receptor. Analysis of NFκB translocation into the nucleus as a measure for NFκB activation showed increased NFκB levels in the nucleus of SCZ microglia-like cells (Fig. 3c, d quantification in e). Enhanced expression of NLRP suggests a contribution of inflammasome assembly in SCZ phenotypes. Inflammasome activation is signified by caspase-1 activation^28^. Measurement of caspase-1 activity as a readout for inflammasome functioning revealed increased caspase-1 activation in SCZ samples in comparison to healthy controls (Fig. 3f). This finding was replicated after stimulation with LPS. We conclude that SCZ microglia-like cells show an increased inflammatory state signified by enhanced inflammasome activity.

### Neurons derived from SCZ-iPSCs exhibit reduced synaptic density

**Fig. 4:**
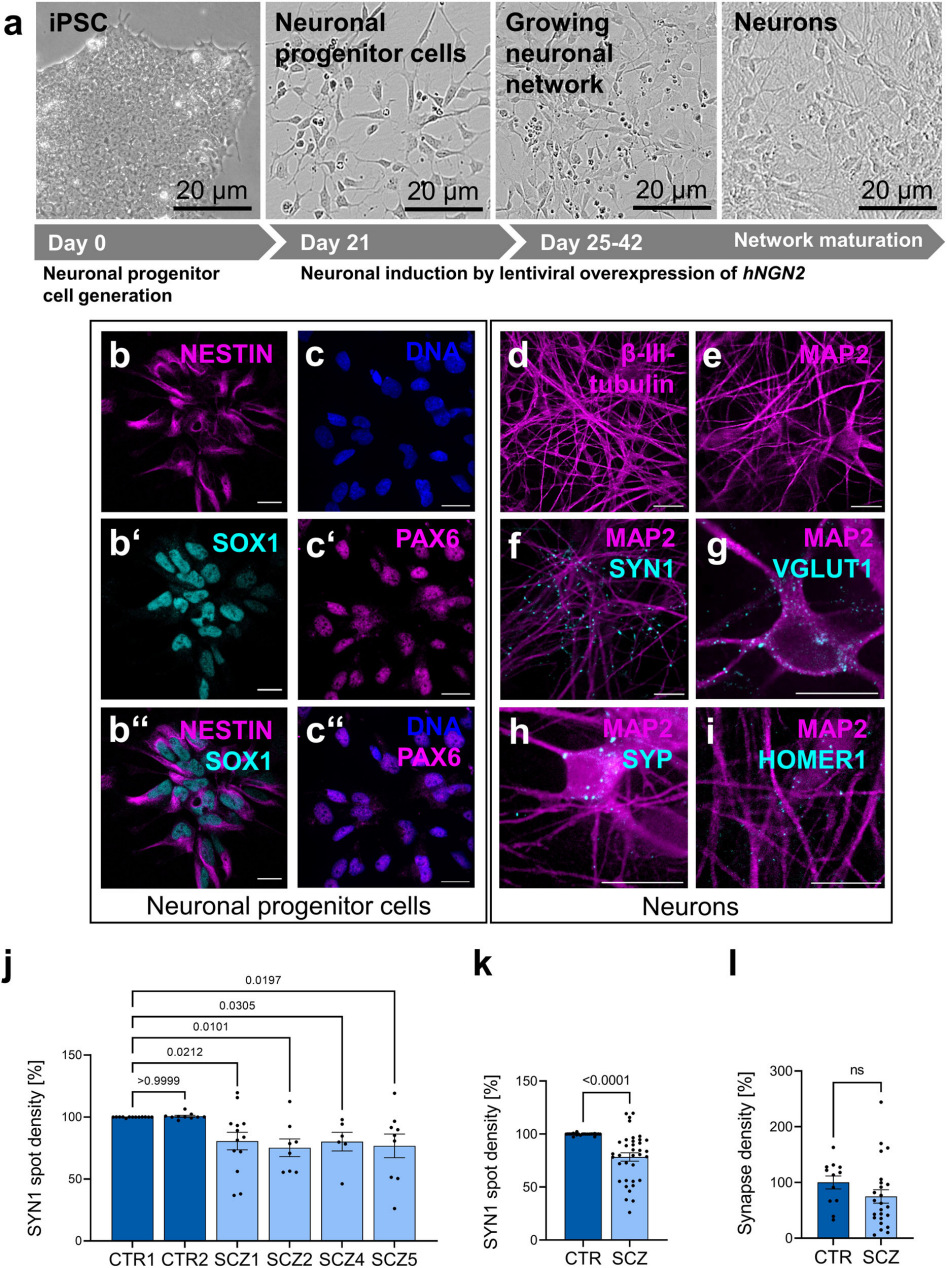
Presynaptic marker density is significantly reduced in neuronal cultures derived from SCZ patients. **a** Schematic overview of neuronal induction and maturation through lentiviral overexpression of hNGN2. Representative images taken at 63x magnification using confocal microscopy and characterization of neuronal progenitor cells by immunocytochemistry with expression of standard markers such as NESTIN (**b**), SOX1 (**b’**, merge of NESTIN and SOX1 in **b”**), Hoechst for DNA (**c**), PAX6 (**c’**, merge of **c** and **c’** in **c”**). Scale bar 20 µm. **d**–**i** Representative images taken at 63x magnification using confocal microscopy and characterization of neuronal networks by immunocytochemical stainings for neuronal markers β-III-tubulin or MAP2 and presynaptic markers Synapsin 1 (SYN1), vesicular glutamate transporter 1 (VGLUT1) and Synaptophysin (SYP) as wells as the postsynaptic marker protein HOMER1. Scale bar 20 µm. **j** Reduced presynaptic density was quantified by analyzing the number of Synapsin 1 spots detected on MAP2-positive neuronal networks. Control clone CTR1 was set as 100%. Kruskal–Wallis test with Dunn’s post hoc test, H(5) = 25.5 (CTR1 vs. CTR2 *p* > 0.9999, CTR1 vs, SCZ1 *p* = 0.0212, CTR1 vs. SCZ2 *p* = 0.0101, CTR1 vs. SCZ4 *p* = 0.0305, CTR1 vs. SCZ5 *p* = 0.0197). Single data points refer to averaged signals obtained from biological replicates. Data are represented as mean ± SEM (CTR1 n = 13; CTR2 n = 10; SCZ1 *n* = 13, SCZ2 *n* = 8; SCZ4 n = 6; SCZ5 *n* = 9), ns=not significant. **k** SYN1 density in pooled control and patient-derived neuronal cultures. Unpaired, two-tailed Mann–Whitney U test. Data are represented as mean ± SEM (*n* = 23 for CTR, *n* = 36 for SCZ), *p* < 0.0001. **l** Synapse density in pooled control and patient-derived neuronal cultures. Unpaired, two-tailed Mann–Whitney U test. Data are represented as mean ± SEM (*n* = 12 for CTR, *n* = 24 for SCZ), ns not significant.

For the setup of a microglia-neuron co-culture model, glutamatergic neurons were differentiated from iPSC-derived neuronal progenitor cells (NPC) by lentiviral overexpression of human Neurogenin 2 (*hNGN2*; Fig. 4a) and further co-cultivated with murine astrocytes to enhance neuronal maturation and synapse formation as published previously (Supplementary Fig. 6^29^). NPCs expressed their cognate marker proteins Nestin, SOX1 and PAX6 (Fig. 4b–b”, c–c”). Differentiated neurons elaborated β-III-tubulin-positive as well as MAP2-positive neurites (Fig. 4d, e), and showed expression of presynaptic (Synapsin 1, Synaptophysin, VGlut1) and postsynaptic (Homer) marker proteins (Fig. 4f–i). Presynapse densities were subsequently calculated as the number of Synapsin 1 (SYN1)-positive spots within MAP2-positive dendritic segments (Fig. 4j, k). The analysis revealed a significant reduction of SYN1 spots in neurons derived from individual patients with SCZ. Data retrieved across experimental replicates demonstrate reproducibility and robustness (Supplementary Fig. 7). Likewise, colocalizing presynaptic and postsynaptic markers as a measure for morphological synapses showed a trend to reduced synapse densities in SCZ samples (Fig. 4l). The results showed a similar outcome as for the analysis of presynaptic markers identified on postsynaptic MAP2-positive dendrites. In conclusion, neurons derived from SCZ patients showed an intrinsic deficit in the formation or maintenance of presynapses.

### Microglia-neuron interactions have a synergistic effect on SCZ-induced neuronal deficits

**Fig. 5:**
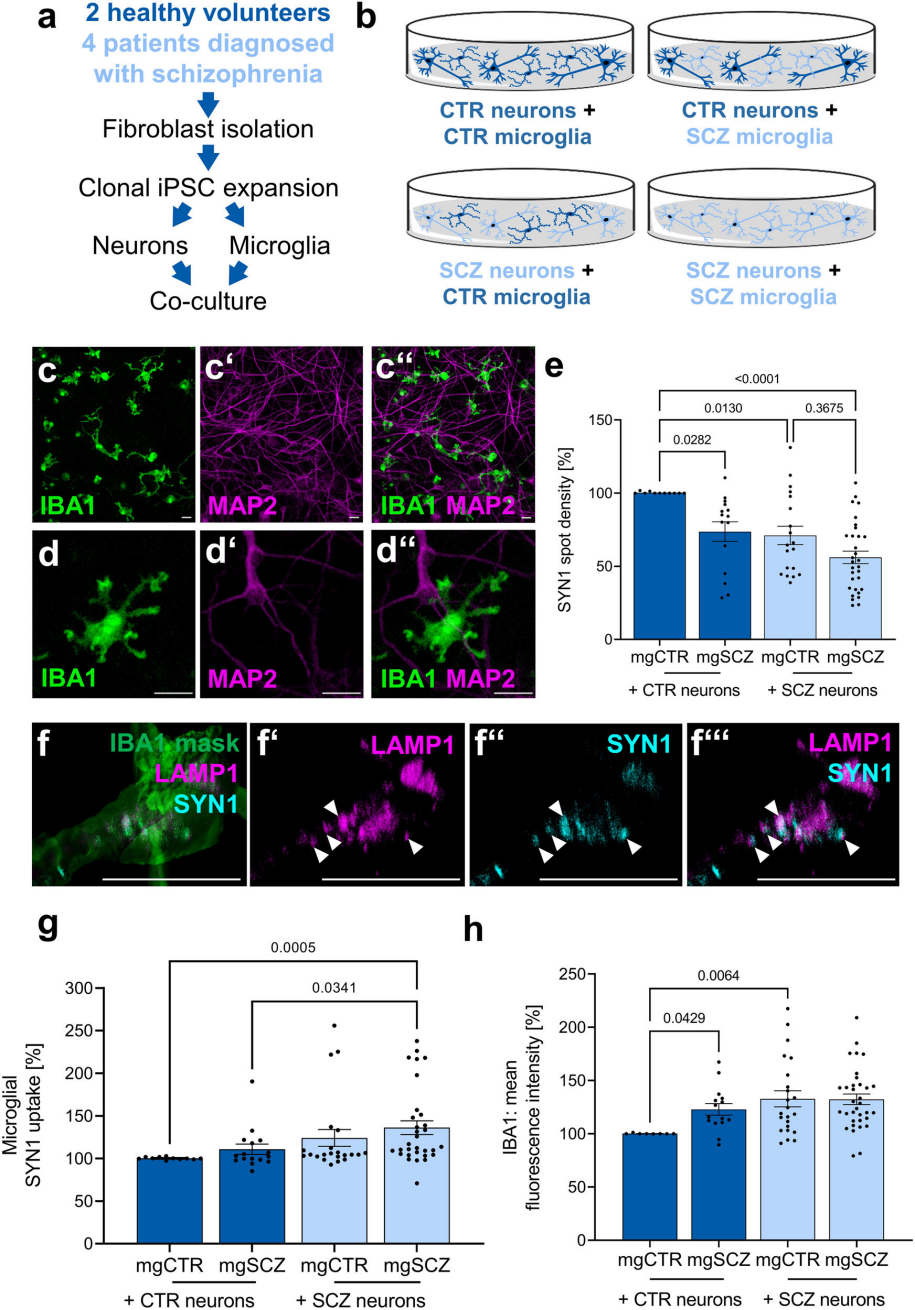
Reciprocal interplay of microglia and neurons in vitro reveals aberrant presynaptic uptake by SCZ microglia. **a** Design of co-culture setup comprising iPSC reprogramming from control- or patient-derived fibroblasts. iPSCs are expanded and differentiated separately towards microglia or neurons. Finally, microglia and neurons are seeded into co-culture at a ratio of approximately 1:5 for 72 h. **b** Schematic overview of the different combinations of control- or patient-derived microglia and neurons used in this study. The image was created using the Illustration Toolkits from Motifolio Inc. **c**, **d** Representative immunocytochemical images taken at x20 or at x63 magnification using confocal microscopy of IBA1 positive microglia in co-culture with MAP2 positive neurons. **c**, **d** IBA1, **c’**, **d’** MAP2**, c”**, **d”** merge of IBA1 and MAP2. Scale bar 20 µm. **e** Density of SYN1 spots was significantly reduced in SCZ microglia-neuron co-cultures. SYN1 spot densities were quantified on MAP2-positive neuronal networks and were normalized to SYN1 spot densities on CTR neurons cultivated in the absence of microglia (Supplementary Fig. 8). Kruskal–Wallis test with Dunn’s post hoc test. H(3) = 23.92 (nCTR+mgCTR vs. nCTR+mgSCZ p = 0.0282, nCTR+mgCTR vs. nSCZ+mgCTR p = 0.0130, nCTR+mgCTR vs. nSCZ+mgSCZ p < 0.0001, nSCZ+mgCTR vs. nSCZ+mgSCZ p = 0.3675). Single data points refer to averaged signals obtained from biological replicates. Data are represented as mean ± SEM, (nCTR+mgCTR1 n = 11; nCTR+mgSCZ n = 15; nSCZ+mgCTR n = 19, nSCZ+mgSCZ n = 30), n=neuron and mg=microglia. **f**–**f”’** Representative images taken at x63 magnification using confocal microscopy of LAMP1 and SYN1 co-localization in IBA1 positive microglia as indicated by white arrow heads. A 3D stack of confocal images after IBA1 staining for microglia served as a mask to identify co-localizing endosomal LAMP1/SYN1 structures. Scale bar 20 µm. **g** Change in mean fluorescence intensity of SYN1 within IBA1 positive microglia was analyzed for quantification of active presynaptic uptake by microglia. Kruskal-Wallis test with Dunn’s post hoc test, H(3) = 18.24 (nCTR+mgCTR vs. nSCZ+mgSCZ p = 0.0005, nCTR+mgSCZ vs. nSCZ+mgSCZ p = 0.0341). Data are normalized to CTR microglia (mgCTR) cultured on CTR neurons (nCTR) and are represented as mean ± SEM, (nCTR+mgCTR1 n = 11; nCTR+ mgSCZ n = 16; nSCZ+mgCTR n = 21, nSCZ+mgSCZ *n* = 31), *n*=neuron and mg=microglia. **h** Microglial activation as quantified by the change of mean IBA1 fluorescence intensity of microglia after co-culture. Kruskal–Wallis test with Dunn’s post hoc test, H(3) =  15.05 (nCTR+mgCTR vs. nCTR+mgSCZ p = 0.0429, nCTR+mgCTR vs. nSCZ+mgCTR p = 0.0064). Data are normalized to CTR microglia (mgCTR) cultured on CTR neurons (nCTR) and are represented as mean ± SEM, (nCTR+mgCTR1 n = 8; nCTR+ mgSCZ n = 15; nSCZ+mgCTR n = 23, nSCZ+mgSCZ n = 34), n, neuron and mg, microglia.

Neurons and microglia-like cells were separately differentiated from iPSC and subsequently combined in co-cultures comprising all four combinations of healthy/SCZ microglia and healthy/SCZ neurons (Fig. 5a, b). After 72 h, IBA1-positive microglia cultivated on MAP2-positive neuronal networks displayed a ramified morphology (Fig. 5c–c”, higher magnification Fig. 5d–d”). For the evaluation of reciprocal interactions between neurons and microglia in SCZ, we examined presynaptic punctae densities of neuronal dendrites (Fig. 5e), the phagocytosis of presynaptic material by microglia (Fig. 5g), and the activation state of microglia (Fig. 5h, Supplementary Fig. 12).

First, presynaptic Synapsin1 (SYN1) spot densities in neurons localized at MAP2-positive dendrites were determined as an approximation for the impact of microglia on neuronal presynaptic punctae numbers (Fig. 5e). The mere addition of microglia of any kind significantly reduced the density of SYN1 spots as compared to a pure neuronal culture (Supplementary Fig. 8), suggesting baseline elimination of presynaptic terminals by microglia-like cells. Addition of SCZ microglia (mgSCZ) to CTR neurons significantly reduced the number of SYN1 spots in comparison to healthy control microglia (mgCTR, Fig. 5e, Supplementary Fig. 9). Likewise, exposure of SCZ microglia to SCZ neurons revealed a trend to further exacerbate the intrinsic presynaptic deficits of SCZ neurons. In the presence of CTR microglia, presynaptic densities of SCZ neurons were significantly decreased in comparison to CTR neurons. This observation is in accordance with the observed intrinsic deficit of SCZ neurons to form presynaptic terminals (Fig. 4j). The stepwise decrease in synapse numbers from the control situation (CTR neurons/CTR microglia) with an intermediate effect for the mixed combinations (SCZ neurons and CTR microglia or CTR neurons and SCZ microglia) towards a maximal loss of presynaptic spots in co-cultures of SCZ neurons and SCZ microglia suggests that presynaptic depletion is a synergistic result of deficient intrinsic presynapse formation in SCZ neurons and aberrant presynapse elimination by SCZ microglia.

Due to the phagocytic activity of microglial cells, we were prompted to investigate whether the presynaptic loss that occurs on dendrites is due to increased uptake of synaptic material by microglia. We therefore analyzed the uptake of presynaptic material into IBA1-positive microglia co-cultured with neurons (Fig. 5f–f”’, g). Uptake of synaptic material was detected and quantified as outlined in Supplementary Fig. 10. Immunocytochemistry revealed co-localization of SYN1-positive material and LAMP1-positive lysosomes suggesting active uptake of presynapses and thus presynaptic pruning by microglia-like cells (Fig. 5f–f”’). Quantification of presynapse uptake (Fig. 5g) showed increasing presynaptic uptake in parallel with decreasing neuronal presynapse density (Fig. 5e) indicating active elimination of presynapses by microglia. Presynapse uptake by SCZ microglia co-cultured with SCZ neurons was significantly increased as compared to CTR microglia with CTR neurons, which is in line with an enhanced microglial phagocytosis in SCZ. SCZ microglia phagocytosed more presynaptic material from SCZ neurons than from CTR neurons, providing further evidence for increased susceptibility of SCZ neurons to a microglial impact beside the intrinsic deficits of SCZ neurons to form presynapses. To understand the impact of soluble factors as a mediator of microglial impact, we next asked the question whether a direct microglia-neuron cell-cell contact was required for presynapse removal. Supernatants from neuron-microglia co-cultures of all combinations were collected and applied to pure neuronal CTR and SCZ cultures. While SYN1 density was generally reduced in SCZ neurons, no impact of cell culture supernatants of any kind was observed (Supplementary Fig. 11) suggesting that cell-cell contacts rather than soluble factors are mediating active presynapse elimination by microglia.

Increased phagocytosis may be explained by an impact of SCZ neurons on microglia. Therefore, we examined expression of the microglial marker IBA1 as a potential measure for a phagocytotic phenotype of microglia activation in neuron-microglia co-cultures^30^. IBA1 expression was quantified in all combinations of co-cultures from CTR and SCZ individuals (Fig. 5h). IBA1 expression was increased in SCZ microglia as compared to CTR microglia after co-culture on CTR neurons, indicating that SCZ microglial cells are intrinsically activated. CTR microglia exposed to SCZ neurons showed an increased activation state compared to CTR microglia that were co-cultured with CTR neurons. This suggests a direct impact of SCZ neurons on microglia resulting in increased levels of microglial activation. Microglial activation was also tested by analysis of CD11b expression as an alternative marker potentially linked to phagocytosis (Supplementary Fig. 12). In conclusion, enhanced expression of IBA1 and CD11b in the presence of increased presynaptic marker uptake suggests microglial activation when exposed to SCZ neurons. Therefore, intrinsic enhanced activation of SCZ microglia may become further increased by extrinsic signals supplied by SCZ neurons.

### Anti-inflammatory pretreatment of microglia rescues SCZ phenotypes

**Fig. 6:**
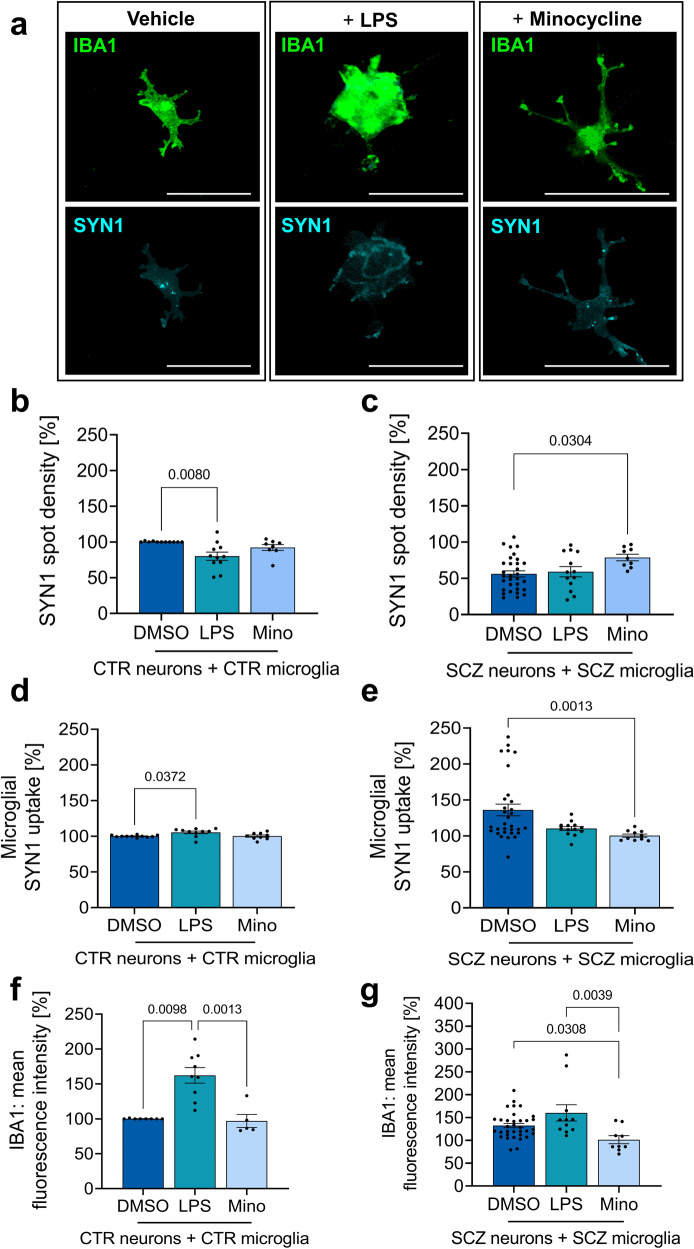
Anti-inflammatory pretreatment of microglia inhibits aberrant presynaptic uptake in SCZ patient-derived microglia. **a** Representative images taken at x63 magnification using confocal microscopy of immunocytochemical staining of SYN1 positive structures within IBA1 positive microglia after LPS (100 ng/ml) or minocycline (10 µM) treatment. Scale bar 20 µm. **b** Quantification of SYN1 spots in control cultures after co-culture with pretreated microglia. SYN1 spot densities were quantified on MAP2-positive neuronal networks. Kruskal-Wallis test with Dunn’s post hoc test, H(2)=9.073 (DMSO vs. LPS p = 0.0080). Single data points refer to averaged signals obtained from biological replicates. Data are represented as mean ± SEM, (DMSO n = 11; LPS n = 11; Mino n = 8). **c** Quantification of SYN1 spots on SCZ neurons exposed to pretreated SCZ microglia. Kruskal–Wallis test with Dunn’s post hoc test, H(2) = 6.692 (DMSO vs. Mino p = 0.0304). Single data points refer to averaged signals obtained from biological replicates. Data are represented as mean ± SEM, (DMSO n = 31; LPS n = 12; Mino n = 8). **d** Analysis of SYN1 uptake by pretreated IBA1-positive CTR-microglia after co-culture with CTR-neurons. SYN1 intensity was quantified in IBA1-positive masks. Kruskal-Wallis test with Dunn’s post hoc test, H(2) = 7.691 (DMSO vs. LPS p = 0.0372). Single data points refer to averaged signals obtained from biological replicates. Data are represented as mean ± SEM, (DMSO n = 11; LPS n = 10; Mino n = 9). **e** Analysis of the mean SYN1 fluorescence intensity within pretreated IBA1 positive SCZ-microglia after co-culture with SCZ-neurons. Kruskal–Wallis test with Dunn’s post hoc test, H(2) = 12.47 (DMSO vs. Mino p = 0.0013). Single data points refer to averaged signals obtained from biological replicates. Data are represented as mean ± SEM, (DMSO n = 31; LPS n = 12; Mino n = 9). **f** Microglial activation as determined by quantification of mean IBA1 fluorescence intensity of pretreated CTR-microglia after co-culture with CTR-neurons. Kruskal-Wallis test with Dunn’s post hoc test, H(2) = 15.14 (DMSO vs. LPS p = 0.0098 and LPS vs. Mino p = 0.0013). Single data points refer to averaged signals obtained from biological replicates. Data are represented as mean ± SEM, (DMSO n = 8; LPS n = 9; Mino n = 5). **g** Microglial activation as determined by quantification of mean IBA1 fluorescence intensity of pretreated SCZ-microglia after co-culture with SCZ-neurons. Kruskal-Wallis test with Dunn’s post hoc test, H(2) = 10.71 (DMSO vs. Mino p = 0.0308 and LPS vs. Mino p = 0.0039). Single data points refer to averaged signals obtained from biological replicates. Data are represented as mean ± SEM, (DMSO n = 34; LPS n = 11; Mino n = 9).

Next, we asked whether modulation of microglial inflammasome functioning mitigates the observed reduction in presynapse densities provoked by neuron-microglia interactions. To this end, we applied two modulators of inflammasome signalling including LPS treatment for the activation of TLR/NFκB/NLRP signaling as well as the antibiotic minocycline as an inflammasome inhibitor. The tetracycline antibiotic minocycline activates Nrf2 and thereby reduces reactive oxygen species-induced inflammasome activation^31^. We pretreated microglial cells with minocycline prior to exposure to neurons in the co-culture model and compared CTR microglia/CTR neuron combinations with SCZ microglia/SCZ neurons (Fig. 6, example images in Fig. 6a). Pretreatment of microglia with minocycline did not modulate neuronal presynapse densities in the control situation (Fig. 6b). However, presynaptic densities were specifically increased in the SCZ co-cultures containing minocycline pretreated SCZ microglia (Fig. 6c). Quantification of microglial presynapse uptake revealed no impact of minocycline in the control setup while in SCZ co-cultures presynapse uptake was significantly decreased (Fig. 6d, e). Accordingly, minocycline reduced microglial activation as measured by IBA1 expression as a potential activation marker only in SCZ co-cultures, while having no effect on CTR microglia further underlining the selective impact of minocycline on SCZ samples (Fig. 6f, g). Interestingly, LPS pretreatment selectively reduced the density of presynapses in CTR neurons (Fig. 6b), and increased uptake of presynaptic material in CTR microglia (Fig. 6d), while no effects were observed in SCZ co-cultures (Fig. 6c+e). In contrast, LPS treatment increased IBA1 expression and microglial activation under CTR conditions (Fig. 6f). Further combinations of control and patient-derived neuron-microglia co-cultures are shown in Supplementary Fig. 13. In conclusion, minocycline rescued the SCZ phenotype regarding microglial activation, presynapse uptake and neuronal presynaptic densities. LPS enhanced microglial activation as suggested by increased IBA1 expression. However, this effect does not precipitate into increased loss of neuronal presynapses or increased microglial uptake of presynaptic material in SCZ samples, suggesting a saturating effect of SCZ-related mechanisms.

## Discussion

Patient-derived and disease-specific cellular models offer great potential to better understand the ambivalent communication of key cellular players in neurodevelopmental disorders with a huge heterogeneity and divergent molecular causes. Here, we present an iPSC-based co-culture model comprising human neurons and microglia to analyze neuro-immune interactions in SCZ samples. We demonstrate that both neuronal and microglial features contribute to excessive elimination of presynaptic terminals. Intrinsic properties of SCZ patient-derived neurons lead to decreased presynaptic densities, while SCZ microglia showed an enhanced activation state of microglia, increased TNFα secretion and elevated NFκB signaling compared to healthy microglia. This was accompanied by an upregulation of inflammasome genes NLRP2 and NLRP3, which are targets of NFκB signaling and are involved in the activation of the inflammasome by caspase-1.

Increased IBA1 immunoreactivity indicates an enhanced activated state of SCZ microglia, which correlates with increased TNFα secretion. In this line, peripheral TNFα is increased in SCZ as found in a meta-analysis of patients with SCZ^32^. TNFα may bind to the TNFα receptor and subsequently induce NFκB signaling. The TNFα gene by itself represents a downstream target of NFκB and may therefore serve for sustained maintenance of an activated microglial state^33^. Our expression analysis also implies that other receptors such as TLR4 become upregulated in SCZ microglia, which may further exacerbate NFκB signaling^34^.

Our observations strongly argue for a contribution of the inflammasome system in SCZ as documented by increased NFκB signaling linked with upregulated NLRP2/3, and enhanced caspase-1 activity. Inflammasome functioning relies on two consecutive steps including priming and activation that finally activate caspase-1 for cleavage and secretion of IL-1β^28^. The priming step includes activation of NFκB and upregulation of inflammasome genes. We observed induction of NLRP2 and NLRP3 transcription both of which represent central components of the inflammasome^35,36^. NLRPs subsequently serve as sensors for cellular stress for inflammasome activation^28^. Mitochondrial dysfunction and production of reactive oxygen species serve as a potential inflammasome activator and are discussed as an important mechanism contributing to SCZ^37–39^. Interestingly, minocycline was shown to inhibit reactive oxygen species-dependent inflammasome activation through stabilization of the antioxidant Nrf2^31,40^. Here, we report that minocycline pretreatment of microglial cells rescued both the activation state of SCZ microglia and additionally preserved neuronal presynapses in the co-culture model. This observation therefore supports the hypothesis that inflammasomes are crucially involved in microglia-dependent synaptic loss in SCZ.

A final step includes the activation of the inflammasome component caspase-1, which is required for cleavage of pro-IL-1β and subsequent release of IL-1β. Increased levels of IL-1β were found in patients with SCZ^41^. In accordance with our observation that presynaptic densities are reduced in neurons upon exposure to SCZ microglia, IL-1β was suggested to inhibit BDNF signaling, a mechanism required for morphological and functional synaptic plasticity^42^. In accordance with an impact on synapses in particular of the prefrontal cortex, minocycline ameliorates negative symptoms in patients with SCZ and reduces IL-1β levels in parallel^43^. It is of note that minocycline selectively improved SCZ phenotypes and did neither modulate presynaptic densities, nor SYN1 uptake or IBA1 expression in microglia of control samples, suggesting a specific effect on SCZ samples not found with healthy controls. Accordingly, a comparable effect of minocycline treatment was also described in a meta-analysis of preclinical studies performed using animal models for depression. Minocycline treatment was only effective with animals that have experienced severe stressful situations before assessment of depressive states, while no effects were observed in testing naïve animals in an otherwise healthy state^44^.

To conclude, deficient synapse formation in iPSC-derived models of SCZ neurons has been documented by several reports^13–15^. In this study, we present a new, fully iPSC-based model of microglia and neurons to study neuro-immune interactions. Our results imply an impact of SCZ neurons on microglia as suggested by increased microglial activation and excessive uptake of presynapses in the presence of SCZ neurons. Vice versa, neurons display reduced presynaptic densities when exposed to SCZ microglia. The precise nature of mutual neuron-microglia interactions remains elusive. However, rescue of SCZ phenotypes by minocycline pretreatment of microglia links our findings to excessive inflammation and IL-1β release. Likewise, deposition of components of the complement system on SCZ presynaptic terminals and interaction with microglial complement systems as also shown by others may provide an explanation for increased susceptibility and concomitant aberrant synaptic pruning^16,45^. In line with this, we have observed increased expression of complement factor C4 in SCZ microglial cells. However, further potential mechanisms of mutual interactions may also apply. The involvement of microglial inflammasome activation points at further extracellular cues to engage TLR activation for induction of NFκB signalling. Such extracellular ligands may comprise extracellular ligands such as Damage-Associated Molecular Patterns (DAMPs) e.g. neuronal High Mobility Group Protein 1 which becomes upregulated in patients with SCZ^46,47^. Mitochondrial dysfunction in SCZ neurons and release of mitochondrial DAMPs may account for further mechanisms of microglial activation^48,49^. Finally, neuroinflammation in SCZ may also rely on disturbed neuronal CX3CL1 interaction with microglial CX3CR1 or compromised Neuregulin signalling^50–52^.

Although differentiation of microglia and neurons within 19 days and a co-culture of three days is not able to adequately represent aberrant neurodevelopment and neuro-immune interaction evolving over several years in human patients, our model overcomes the need for non-human co-culture models and retains patient-specific phenotypes. Here we recapitulate SCZ features of reduced presynaptic density and increased microglial activation as previously visualized by PET imaging, in postmortem studies or rodent models^53,54^. It will be interesting in the future to additionally test minocycline in microglia-neuron co-culture models derived from larger cohorts of iPSCs with different genetic backgrounds and in unaffected siblings of SCZ patients.

In summary, our iPSC-derived and patient-specific co-culture system offers the opportunity to study neuron-microglia interactions in SCZ in more detail. The specific effect of minocycline on SCZ microglia represents a promising approach for adjunctive therapy to antipsychotic treatment and may be helpful for future drug development.

## Material and methods

The methods were performed in accordance with relevant guidelines and regulations and approved by the Ethics Committee of the University Hospital and Faculty of Medicine Tuebingen. We confirm that participants provided a written informed consent to take part in the study. Inclusion and exclusion criteria for the selection of patients diagnosed with SCZ are described in Supplementary Table 1. iPSCs were generated and fully characterized as described elsewhere (Table 1; refs. ^22,23^). All experiments were carried out with all lines in parallel.

### Study design, iPSC reprogramming and maintenance

Patient-derived human fibroblasts were reprogrammed by nucleofection of non-integrative, episomal vectors encoding for OCT3/4, SOX2, LIN28, KLF4, c-MYC, p53 and EBNA1 (Addgene, catalog no. 41813, 41814, 41855, 41856, 41857). Electroporated fibroblasts were seeded onto Matrigel (Corning, catalog no. 354277) coated well plates and expanded in a feeder-free culture system for 21–28 days until first iPS colonies appeared. iPS clones were manually picked and expanded on Matrigel in mTeSR Plus medium (STEMCELL Technologies, catalog no. 05825). Passaging was routinely performed non-enzymatically using Gentle Cell Dissociation Reagent (STEMCELL Technologies, catalog no. 100-0485) in early passage numbers or later enzymatically by using accutase (Sigma Aldrich, catalog no. A6964). All expanded iPS clones were routinely tested for expression of stem cell marker on protein and RNA level and pluripotency. All iPS clones used in this study were chromosomally intact.

### Microglia differentiation

For microglia differentiation, we modified a previously published protocol for the differentiation of iPSC into monocytes and macrophages^55^. iPSCs were dissociated using accutase and seeded at a density of 5 × 10^4^ cells per cm² (day -2). Only wells containing equally distributed iPSC colonies of 10–20 cells were considered for differentiation. For all differentiation steps, a 1:1 mix of IMDM without phenol red (Thermo Fisher Scientific, catalog no. 21056023) and Ham’s F12 Nutrient Mix (Thermo Fisher Scientific, catalog no. 21765029) was used. The basal medium was supplemented with 10 µg/mL poly vinyl alcohol (Sigma Aldrich, catalog no. P8136), 64 µg/mL ascorbic acid 2 phosphate (Sigma Aldrich, catalog no. A8960), 0.1x chemically defined lipid concentrate (Thermo Fisher Scientific, catalog no. 11905031), 2x ITS-X (Thermo Fisher Scientific, catalog no. 51500056), 0.0039% α-Monothioglycerol (Sigma Aldrich, catalog no. M6145), 1x GlutaMAX (Thermo Fisher Scientific, catalog no. 35050061) and 1x non-essential amino acids. Additional growth factors and cytokines were always added freshly before usage. For mesoderm induction at day 0, the basal medium was supplemented with 50 ng/mL BMP4 (Peprotech, catalog no. 120-05ET), 15 ng/mL Activin A (Miltenyi Biotec, catalog no. 130-115-008) and 1.5 µM CHIR99021 (Axon Medchem, catalog no. 1386) for mesoderm induction. For the suppression of self-renewal in favor of stem cell differentiation at day 2, 10 µM of SB431542 and SCF (Peprotech, catalog no. 300-07), VEGF (Peprotech, catalog no. 100-20) and bFGF (Bio-Techne, catalog no. 233-FB) were added to the medium at a final concentration of 50 ng/ml each. For hematopoietic patterning at day 5, 10 ng/ml of IL-3 (Peprotech, catalog no. 200-03) and 50 ng/ml of IL-6 (Peprotech, catalog no. 200-06), 50 ng/ml of TPO (Miltenyi Biotec, catalog no. 130-095-747), 50 ng/ml of bFGF, 50 ng/ml of SCF and 50 ng/ml of VEGF were supplemented to the basal medium. Medium was refreshed at day 7. At day 9, differentiated cells grew to full confluence with hematopoietic stem cells emerging into the supernatant. Adherent cells were dissociated by accutase treatment and added to non-adherent cells collected from the supernatant. After centrifugation at 300 x *g* for 3 min, cells were resuspended in microglia medium containing 100 ng/ml of IL-34 (Peprotech, catalog no. 200-34), 50 ng/ml of TGFβ-1 (Peprotech, catalog no. 100-21) and 25 ng/ml of GM-CSF (Peprotech, catalog no. 300-03). Cells were subsequently plated on ultra-low attachment plates that were pretreated with Anti-Adherence Rinsing Solution for at least 5 min and afterwards rinsed twice with DPBS. Microglia differentiation was allowed to proceed for further seven days with medium changes every other day.

Microglia were routinely characterized regarding expression of key markers like IBA1, SPI1 and TMEM119. Functionality was proven by active uptake of pHrodo-labelled bacteria and response to LPS as a pro-inflammatory stimulus. Microglia identity was confirmed by RNA sequencing. Transcriptome analysis and bioinformatical evaluation was performed by CeGaT GmbH (Germany) as previously described^15^. For characterization of microglial phenotypes, microglial genes were chosen according to previously published literature that identified panels of highly specific microglia signature genes^21,56–60^.

### ELISA

Secretion of the pro-inflammatory cytokine TNFα was quantified by a standard sandwich-ELISA (human TNF-alpha DuoSet ELISA kit, R&D Systems, catalog no. DY210) according to the manufacturer’s instructions. Briefly, 96-well plates were coated with the capture antibody and incubated over night at room temperature. Wells were washed three times and blocked for at least 1 h at room temperature. Wells were again washed and 100 µl of culture supernatant or standards were added and incubated for 2 h at room temperature. After washing, detection antibody was added and incubated at room temperature for 2 h. Wells were washed and the streptavidin / horse radish peroxidase (HRP) mix was added for 30 min at room temperature. Afterwards, wells were washed again and substrate solution was added for 20 min at room temperature in the dark, stop solution was added and the plate was tapped for mixing. Immediately afterwards, the optical density was determined using a microplate reader (Tecan Spark) set to 450 nm with wavelength corrections set to 540 nm.

### Flow cytometry

Flow cytometry measurements were performed using BD FACS Chorus software on a BD FACS Melody and analyzed using FlowJo 10.6.1 (FlowJo Engine, Becton Dickinson & Company). Cells were detached, washed three times with DPBS and stained with conjugated antibodies for 30 min at 4 °C. Subsequently, cells were washed three times with DPBS and resuspended in PBS + 1% FCS for immediate analysis. The following conjugated antibodies were used: anti-human SSEA-4 PE-Vio770 (Miltenyi Biotech, catalog no. 130-105-081), anti-human CD11b FITC (Thermo Fisher Scientific, catalog no. 11-0118-42) and anti-human CD45 VioBlue (Miltenyi Biotech, catalog no. 130-110-775). Doublets were excluded in FSC and SSC. Unstained cells served as negative population.

### Quantification of p65 expression

Day 19 microglia were plated at a density of 1 × 10^5^ cells/cm² on Matrigel-coated 96 well plates. Cells adhered within 24 h and were subsequently fixed and stained against NFκB p65 (Cell Signaling, catalog no. 6956 T), Phalloidin CruzFluor™ 488 Conjugate (Santa Cruz Biotechnology, catalog no. sc-363791) and Hoechst (Sigma-Aldrich, catalog no. 911004450). Using confocal laser scan microscopy with a 63x plan-apochromatic oil immersion objective, at least ten 3D Z-stacks were acquired of microglia were taken within each experiment. During acquisition, all settings such as exposure time, laser intensity and gain were kept constant. Z-stacks were further processed using Imaris software (Bitplane, version 8.2.0). Therefore, a surface for the nucleus was generated covering the Hoechst signal. Within this mask, the mean fluorescence intensity of NFκB p65 was determined and quantified.

### Caspase-1 activity

Caspase-1 activity was determined using the Caspase-Glo® 1 Inflammasome Assays (Promega, catalog no. G9951) according to the manufacturer’s instructions. Briefly, day 19 microglia were plated at a density of 1.2 × 10^5^ cells/cm² on Matrigel-coated 96 well plates and incubated overnight. The next day, cells were treated with 100 ng/ml of LPS (Sigma Aldrich, catalog no. L6529) for 3 h and subsequently with 5 mM ATP (Sigma Aldrich, catalog no. A2383) for 30 min at 37 °C and 5% CO_2_. The culture supernatant was transferred into a white 96 well plate and Caspase-Glo® 1 Reagent was added. The mixture was incubated at room temperature in the dark for 1 h and luminescence was measured on a Tecan Spark microplate reader.

### Phagocytosis assay

Day 19 microglia and naïve iPSC as control were plated at a density of 2 × 10^4^ cells per well of a 96 well plate and cells adhered within 24 h. pHrodo™ Red *E. coli* BioParticles™ (Thermo Fisher Scientific, catalog no. P35361) were resuspended in 2 ml PBS to generate a stock suspension with a concentration of 1 mg/ml. Bioparticles were vortexed rigorously to generate a homogenous suspension. 10 µl of pHrodo™ Red *E. coli* BioParticles™ were added to the wells. Cells were incubated at 37 °C and 5% CO_2_ for 4 h in the Incucyte® S3 live-cell imaging system (Sartorius). 9 images per well were acquired every 15 min at x20 magnification. Finally, the relative red fluorescent units per image were analyzed over time.

### Lentivirus production

For lentivirus production, HEK293FT were cultured at 37 °C and 8 % CO_2_ in culture medium consisting of DMEM (Thermo Fisher Scientific, catalog no. 10566016), 10% FCS (Thermo Fisher Scientific, catalog no. 10270106), 500 µg/ml G418 (Carl Roth, catalog no. 2039), 1% non-essential amino acids (Thermo Fisher Scientific, catalog no. 11140035) and passaged using 0.25% Trypsin/EDTA (Thermo Fisher Scientific, catalog no. 25200056) once or twice a week. For lentivirus production, cells were dissociated and seeded at a density of 3000 cells per cm². After four days of incubation, medium of HEK293FT cells was changed to a serum-reduced transfection medium of Opti-MEM (Thermo Fisher, catalog no. 11058021) supplemented with 5% FCS. 27 µg of pC-Pack2 Lentiviral Packaging Mix (Cellecta, catalog no. CPCP-K2A) were mixed with 108 µl of Lipofectamine 2000 Reagent (Thermo Fisher Scientific, catalog no. 11668019) in 4.5 mL Opti-MEM, incubated at room temperature for 20 min and added to the cells for further incubation at 37 °C and 5% CO2. After 24 h, medium was changed, while after 48 h and 72 h post-transfection the supernatant was removed and stored at −80 °C. Lentiviral suspensions were filtered through a 22 nm filter, transferred into ultracentrifugation buckets and centrifuged at 19,600 rpm and 4 °C for 80 min. Pellets were air dried for a few minutes and remaining liquid was removed with sterilized soft tissue papers. Finally, 100 μl of DPBS + 1% BSA were added per tube without pipetting or resuspending. Tubes were sealed with Parafilm® and left overnight at 4 °C. The next day, pellets were resuspended by pipetting several times and aliquoted for storage at −80 °C. Titer determination was performed using the Lenti-X p24 Rapid Titer Kit (Takara Bio, catalog no. 632200) according to the manufacturer’s instructions. Lentiviral suspensions were diluted 10-fold and 100-fold and quantified against a p24 standard curve. Yields ranged from 5 × 10^10^ to 5 × 10^11^ particles/ml.

### Neuronal progenitor cell generation

Ectodermal patterning was induced using the STEMdiff™ Neural Induction Kit (STEMCELL Technologies, catalog no. 05835) according to the manufacturer’s instructions. iPSC were dissociated using accutase and 2 × 10^6^ iPSC were seeded into ultra-low attachment AggreWell 800™ well plates (STEMCELL Technologies, catalog no. 34815) pretreated with Anti-Adherence Rinsing Solution (STEMCELL Technologies, catalog no. 07010). After cultivation at 37 °C and 5% CO_2_ for seven days with daily medium changes, embryoid bodies were harvested using 37 µm reversible strainers (STEMCELL Technologies, catalog no. 27215). Prior to seeding, 6-well plates were pretreated with 20% poly-L-ornithine (PLO, Sigma-Aldrich, catalog no. P4957) in Dulbecco’s phosphate-buffered saline (Thermo Fisher Scientific, catalog no. 14190094), incubated for 2 h at room temperature and washed three times with DMEM/F12 (Thermo Fisher Scientific, catalog. no. 21331020). Subsequently, wells were treated with 10 µg/ml laminin (Lam, Sigma-Aldrich, catalog no. L2020) diluted in DMEM/F12 and incubated overnight at 37 °C and 5% CO2. Harvested embryoid bodies were washed to remove remaining single cells and seeded onto PLO/Lam pre-coated well plates in STEMdiff™ Neural Induction Medium with daily medium changes. Neural rosettes were selected using the STEMdiff™ Neural Rosette Selection Reagent (STEMCELL Technologies, catalog no. 05832), resuspended in STEMdiff™ Neural Induction Medium supplemented with 1 µM Dorsomorphin dihydrochloride (Bio-Techne, catalog no. 3093), 10 µM SB 431542 (Bio-Techne, catalog no. 1614), 500 ng/ml recombinant Human Noggin Fc Chimera Protein (Bio-Techne, catalog no. 719-NG) and cultivated in PLO/Lam coated 6-well plate. After the first passage, cultivation medium was changed to STEMdiff™ Neural Progenitor Medium (STEMCELL Technologies, catalog no. 05833). NPCs were passaged up to passage 10. All generated NPCs were routinely tested for progenitor marker expression, such as PAX6, NESTIN or SOX1.

### Neuronal differentiation

Neuronal differentiation was achieved by lentiviral overexpression of human Neurogenin 2 following previously published protocols^61,62^. 3.15 × 10^4^ NPC were dissociated by accutase treatment and seeded in PLO/Lam-coated well plates at a density of 3 × 10^4^ cells per cm² in STEMdiff™ Neural Progenitor Medium. For induction of neuronal differentiation^62^, NPC were co-infected with lentiviral vectors pLV-TetO-hNGN2-Puro (Addgene, catalog no. 79049), and FUdeltaGW-rtTA (Addgene, catalog no. 19780) at a final concentration of approximately 10 ng/ml or 2 × 10^8^ particles/ml per lentivirus. After 24 h, doxycycline (Sigma Aldrich, catalog no. D9891) was added to a final concentration of 10 µg/ml to induce tetracycline-dependent expression of the reverse tetracycline transactivator (rtTA) and hNGN2. 24 h later, 2 µg/ml of puromycine (Thermo Fisher Scientific, catalog no. 11113803) was added to the medium to select for transduced NPC. After removal of selection medium at day 2 post transduction, cells were supplied with neuronal differentiation medium consisting of Neurobasal Plus Medium (Thermo Fisher Scientific, catalog no. A3582901) supplemented with 1x B27 Plus supplement (Thermo Fisher Scientific, catalog no. A3582801), 1x N2 supplement (Thermo Fisher Scientific, catalog no. 17502048), 1 µg/ml Laminin, 20 ng/mL BDNF (Peprotech, catalog no. 450-02), 20 ng/mL GDNF (Peprotech, catalog no. 450-10), 500 µg/mL dibutyryl cyclic adenosine monophosphate (Sigma Aldrich, catalog no. D0627), 35 µg/mL L-Ascorbic Acid (Sigma Aldrich, catalog no. A2078) and 10 μg/ml doxycycline. At this point, 3 × 10^4^ murine primary astrocytes per cm² were added a 50% medium change was performed every other day until neurons were assayed or fixed after 14–21 days in vitro.

### Neuron-microglia co-culture

Neuronal and microglial differentiation started separately from each other for 16 days. Subsequently, microglia were lifted from the ultra-low attachment plates, washed with DPBS, centrifuged and finally resuspended in microglia medium. In case of pretreatment, microglia were primed using 100 ng/ml of LPS or 10 µM of Minocycline (STEMCELL Technologies, catalog no. 74112) at 37 °C and 5% CO_2_ for 60 min. Subsequently, microglial cells were washed with DPBS and added to the neuronal cultures. For a final microglia:neuron ratio of approximately 1:5, microglia were seeded at a density of 5 × 10^4^ microglial cells per cm² combined with 3 × 10^4^ initially seeded NPCs per cm². The co-culture plate was transferred to the incubator and left for 72 h at 37 °C and 5% CO2. Co-cultures were maintained in microglia medium throughout the experiments.

### Immunocytochemistry

iPSC-derived neurons and microglia, cultured in 96-well µclear plates (Greiner Bio, catalog no. 655090), were fixed using paraformaldehyde (4% in PBS, Sigma Aldrich, catalog no. P6148) for 15 min at room temperature. After fixation, cells were washed three times with PBS and then blocked and permeabilized at room temperature in 0.1% Triton X-100/PBS containing 1X Blocking Reagent for ELISA (Merck, catalog no. 11112589001) for 30 min. After overnight incubation at 4 °C with primary antibodies diluted in blocking solution, cells were washed three times in PBS and exposed to fluorescently labeled secondary antibodies (1:500; Cy3 anti-rabbit (Jackson ImmunoResearch, catalog no. 111-165-144) or Cy5-coupled goat anti-mouse secondary antibodies (Jackson ImmunoResearch, catalog no. 115-175-146) and Alexa Fluor® 488-coupled goat anti-chicken or 647-coupled goat anti-rat antibodies (Thermo Fisher Scientific, catalog no. A21247, A11039). Secondary antibodies were dissolved in blocking solution and incubated at room temperature for 2 h. Nuclei were stained using Hoechst Dye 33258 (1:1,000 in PBS, Sigma-Aldrich, catalog no. 911004450). The following primary antibodies were used: mouse monoclonal anti-Beta-Tubulin III (STEMCELL Technologies, catalog no. 60100, 1:250), mouse monoclonal CX3CR1 (BioLegend, catalog no. 355701, 1:500), rabbit polyclonal anti-IBA1 (FUJIFILM Wako Chemicals, catalog no. 019-19741, 1:1000), CD11b monoclonal antibody (ICRF44), eBioscience™ (#14-0118-82), rat monoclonal anti-LAMP1 (Santa Cruz Biotechnology, catalog no. sc-19992, 1:100), chicken polyclonal anti-MAP2 (Invitrogen, catalog no. PA1-10005; 1:2500), mouse monoclonal anti-NFκB p65 (Cell Signaling, catalog no. 6956), mouse monoclonal anti-PAX6 (BioLegend, catalog no. 862001, 1:200), Phalloidin CruzFluor# 488 (Santa Cruz Biotechnology, catalog no. sc-363791), rabbit monoclonal recombinant anti-PSD95 (Synaptic Systems, catalog no. 124008, 1:500), mouse monoclonal anti-SPI1 (PU.1, BioLegend, catalog no. 658002, 1:100), rabbit polyclonal anti-SOX1 (Abcam, catalog no. ab22572, 1:500), mouse monoclonal anti-Synapsin1 (Synaptic Systems, catalog no. 106011, 1:1000), rabbit polyclonal anti-Synaptophysin1 (Synaptic Systems, catalog no. 101002, 1:500), rabbit anti-TMEM119 (Synaptic Systems, catalog no. 400002, 1:400), rabbit monoclonal anti-TREM2 (Cell Signaling, catalog no. 91068, 1:400), mouse monoclonal anti-VGlut1 (Synaptic Systems, catalog no. 135511, 1:300), mouse monoclonal anti-Nestin (Synaptic System, catalog no. 312011, 1:1000). Antibody specificity was confirmed by analysis on differentiated cells and naïve iPSC, and by secondary antibody only stainings.

### Quantification of synaptic marker densities and synaptic uptake

To determine microglial pruning of synaptic structures, Z-stacks of neuronal networks were acquired with a confocal laser scan microscopy Cell Observer SD with a x63 plan-apochromatic oil immersion objective. Z-stacks were retrieved from regions of comparable fibre density, while the settings for acquisition (such as exposure time, laser intensity and gain) were unchanged for all conditions. Each image is a 3D reconstruction of a z-stack.

Images of neuronal cultures or neuron-microglia co-cultures were further processed by imaging using Imaris software. A surface was generated covering all MAP2 signals present in the whole stack. Next, the surface was masked using the Synapsin 1 (SYN1) signal creating a new channel for SYN1. After spot detection in the new SYN1 channel, SYN1-positive synaptic structures were counted after thresholding and referred to the volume of MAP2-positive structures to provide the density of SYN1-positive presynaptic terminals. The threshold for SYN1 spot detection was kept constant for each replicate. Data from multiple images were averaged to give yield to one datapoint for each biological replicate. The number of biological replicates is indicated in the figure legends. Within each image 2-3 microglial cells were analyzed on average. Within individual biological replicates, samples were normalized to the mean of CTR1.

Microglial uptake of synaptic structures was quantified by determination of the mean fluorescence intensity of SYN1 within IBA1 positive microglia. To this end, Z-stacks of microglia were acquired as described above and further processed using Imaris. A first surface was generated using the IBA1 signal to cover whole microglial cells and was subsequently masked with the signal for SYN1. Mean fluorescence intensities were measured for SYN1-positive spots identified within microglia. At least three independent experiments were performed for each donor combination.

### Statistics and reproducibility

Statistical analysis was performed using GraphPad Prism 9.2.0 (GraphPad Software Inc.). For non-Gaussian distribution in pairwise comparisons, the unpaired Mann–Whitney U test was performed and for group comparisons, Kruskal–Wallis test with Dunn’s post-hoc multiple comparisons test was used. The type of statistical tests used and results are reported in the figure legends or main text.

### Reporting summary

Further information on research design is available in the Nature Portfolio Reporting Summary linked to this article.

## Supplementary information

Supplementary Information

Description of Additional Supplementary Files

Supplementary data 1

Reporting Summary

## Data Availability

RNA sequencing data are available for download from the NCBI Gene Expression Omnibus (GEO) (NCBI GEO no. GSE213232). Source data are provided as supplementary information (supplementary data 1). Additional data supporting the findings of this study are available from the corresponding author upon request.

## References

[1] Stefansson H (2009). Common variants conferring risk of schizophrenia. Nature.

[2] Brown AS, Derkits EJ (2010). Prenatal infection and schizophrenia: a review of epidemiologic and translational studies. Am. J. Psychiatry..

[3] Khandaker GM, Zimbron J, Dalman C, Lewis G, Jones PB (2012). Childhood infection and adult schizophrenia: a meta-analysis of population-based studies. Schizophr. Res..

[4] Schizophrenia Working Group of the Psychiatric Genomics, C. (2014). Biological insights from 108 schizophrenia-associated genetic loci. Nature.

[5] Andreassen OA (2015). Genetic pleiotropy between multiple sclerosis and schizophrenia but not bipolar disorder: differential involvement of immune-related gene loci. Mol. Psychiatry..

[6] Goldsmith DR, Rapaport MH, Miller BJ (2016). A meta-analysis of blood cytokine network alterations in psychiatric patients: comparisons between schizophrenia, bipolar disorder and depression. Mol. Psychiatry..

[7] Fillman SG (2016). Elevated peripheral cytokines characterize a subgroup of people with schizophrenia displaying poor verbal fluency and reduced Broca’s area volume. Mol. Psychiatry..

[8] Trepanier MO, Hopperton KE, Mizrahi R, Mechawar N, Bazinet RP (2016). Postmortem evidence of cerebral inflammation in schizophrenia: a systematic review. Mol. Psychiatry..

[9] Glantz LA, Lewis DA (2000). Decreased dendritic spine density on prefrontal cortical pyramidal neurons in schizophrenia. Arch. Gen. Psychiatry..

[10] Borgwardt SJ (2008). Reductions in frontal, temporal and parietal volume associated with the onset of psychosis. Schizophr. Res..

[11] Glausier JR, Lewis DA (2013). Dendritic spine pathology in schizophrenia. Neuroscience.

[12] Osimo EF, Beck K, Reis Marques T, Howes OD (2019). Synaptic loss in schizophrenia: a meta-analysis and systematic review of synaptic protein and mRNA measures. Mol. Psychiatry..

[13] Brennand KJ (2011). Modelling schizophrenia using human induced pluripotent stem cells. Nature.

[14] Wen Z (2014). Synaptic dysregulation in a human iPS cell model of mental disorders. Nature.

[15] Grunwald LM (2019). Comparative characterization of human induced pluripotent stem cells (hiPSC) derived from patients with schizophrenia and autism. Transl. Psychiatry..

[16] Sellgren CM (2019). Increased synapse elimination by microglia in schizophrenia patient-derived models of synaptic pruning. Nat. Neurosci..

[17] Park GH (2020). Activated microglia cause metabolic disruptions in developmental cortical interneurons that persist in interneurons from individuals with schizophrenia. Nat. Neurosci..

[18] Muffat J (2016). Efficient derivation of microglia-like cells from human pluripotent stem cells. Nat. Med..

[19] Douvaras P (2017). Directed Differentiation of Human Pluripotent Stem Cells to Microglia. Stem Cell Reports..

[20] Abud EM (2017). iPSC-Derived Human Microglia-like Cells to Study Neurological Diseases. Neuron.

[21] Butovsky O (2014). Identification of a unique TGF-beta-dependent molecular and functional signature in microglia. Nat. Neurosci..

[22] Stock R (2020). Generation and characterization of human induced pluripotent stem cells lines from four patients diagnosed with schizophrenia and one healthy control. Stem Cell Res..

[23] Keller AL (2021). Generation and characterization of the human induced pluripotent stem cell line NMIi010-A from peripheral blood mononuclear cells of a healthy 49-year old male individual. Stem Cell Res..

[24] de Rivero Vaccari JP, Dietrich WD, Keane RW (2014). Activation and regulation of cellular inflammasomes: gaps in our knowledge for central nervous system injury. J. Cereb. Blood Flow Metab..

[25] Kano, S. I. et al. Glutathione S-transferases promote proinflammatory astrocyte-microglia communication during brain inflammation. *Sci. Signal.* **12**10.1126/scisignal.aar2124 (2019). 10.1126/scisignal.aar2124 PMC6637164 30783009

[26] Pitale PM, Howse W, Gorbatyuk M (2017). Neuronatin Protein in Health and Disease. J. Cell Physiol..

[27] Sekar A (2016). Schizophrenia risk from complex variation of complement component 4. Nature.

[28] Swanson KV, Deng M, Ting JP (2019). The NLRP3 inflammasome: molecular activation and regulation to therapeutics. Nat. Rev. Immunol..

[29] Schildge S, Bohrer C, Beck K, Schachtrup C (2013). Isolation and culture of mouse cortical astrocytes. J. Vis. Exp..

[30] Imai Y, Kohsaka S (2002). Intracellular signaling in M-CSF-induced microglia activation: role of Iba1. Glia.

[31] Shahzad K (2016). Stabilization of endogenous Nrf2 by minocycline protects against Nlrp3-inflammasome induced diabetic nephropathy. Sci. Rep..

[32] Momtazmanesh S, Zare-Shahabadi A, Rezaei N (2019). Cytokine Alterations in Schizophrenia: An Updated Review. Front Psychiatry..

[33] Pekalski J (2013). Spontaneous NF-kappaB activation by autocrine TNFalpha signaling: a computational analysis. PLoS One..

[34] Kawai T, Akira S (2007). Signaling to NF-kappaB by Toll-like receptors. Trends. Mol. Med..

[35] Fontalba A, Gutierrez O, Fernandez-Luna JL (2007). NLRP2, an inhibitor of the NF-kappaB pathway, is transcriptionally activated by NF-kappaB and exhibits a nonfunctional allelic variant. J. Immunol..

[36] Bauernfeind FG (2009). Cutting edge: NF-kappaB activating pattern recognition and cytokine receptors license NLRP3 inflammasome activation by regulating NLRP3 expression. J. Immunol..

[37] Do KQ, Cabungcal JH, Frank A, Steullet P, Cuenod M (2009). Redox dysregulation, neurodevelopment, and schizophrenia. Curr. Opin. Neurobiol..

[38] Steullet P (2016). Redox dysregulation, neuroinflammation, and NMDA receptor hypofunction: A “central hub” in schizophrenia pathophysiology?. Schizophr. Res..

[39] Cuenod M (2021). Caught in vicious circles: a perspective on dynamic feed-forward loops driving oxidative stress in schizophrenia. Mol. Psychiatry..

[40] Liu X (2017). Nuclear Factor E2-Related Factor-2 Negatively Regulates NLRP3 Inflammasome Activity by Inhibiting Reactive Oxygen Species-Induced NLRP3 Priming. Antioxid Redox Signal..

[41] Bishop JR, Zhang L, Lizano P (2022). Inflammation Subtypes and Translating Inflammation-Related Genetic Findings in Schizophrenia and Related Psychoses: A Perspective on Pathways for Treatment Stratification and Novel Therapies. Harv. Rev. Psychiatry..

[42] Tong L (2012). Brain-derived neurotrophic factor-dependent synaptic plasticity is suppressed by interleukin-1beta via p38 mitogen-activated protein kinase. J. Neurosci..

[43] Zhang L (2019). The effect of minocycline on amelioration of cognitive deficits and pro-inflammatory cytokines levels in patients with schizophrenia. Schizophr. Res..

[44] Reis DJ, Casteen EJ, Ilardi SS (2019). The antidepressant impact of minocycline in rodents: A systematic review and meta-analysis. Sci. Rep..

[45] Stevens B (2007). The classical complement cascade mediates CNS synapse elimination. Cell.

[46] Yang, H. et al. HMGB1 released from nociceptors mediates inflammation. *Proc. Natl. Acad. Sci.* **118**10.1073/pnas.2102034118 (2021). 10.1073/pnas.2102034118 PMC8379951 34385304

[47] Al-Dujaili AH, Mousa RF, Al-Hakeim HK, Maes M (2021). High Mobility Group Protein 1 and Dickkopf-Related Protein 1 in Schizophrenia and Treatment-Resistant Schizophrenia: Associations With Interleukin-6, Symptom Domains, and Neurocognitive Impairments. Schizophr Bull..

[48] Clay HB, Sillivan S, Konradi C (2011). Mitochondrial dysfunction and pathology in bipolar disorder and schizophrenia. Int. J. Dev. Neurosci..

[49] Bajwa E, Pointer CB, Klegeris A (2019). The Role of Mitochondrial Damage-Associated Molecular Patterns in Chronic Neuroinflammation. Mediators Inflamm..

[50] Bergon A (2015). CX3CR1 is dysregulated in blood and brain from schizophrenia patients. Schizophr. Res..

[51] Corfas G, Roy K, Buxbaum JD (2004). Neuregulin 1-erbB signaling and the molecular/cellular basis of schizophrenia. Nat. Neurosci..

[52] Calvo M (2010). Neuregulin-ErbB signaling promotes microglial proliferation and chemotaxis contributing to microgliosis and pain after peripheral nerve injury. J. Neurosci..

[53] Bloomfield PS (2016). Microglial Activity in People at Ultra High Risk of Psychosis and in Schizophrenia: An [(11)C]PBR28 PET Brain Imaging Study. Am. J. Psychiatry..

[54] Onwordi EC (2020). Synaptic density marker SV2A is reduced in schizophrenia patients and unaffected by antipsychotics in rats. Nat. Commun..

[55] Cao X, van den Hil FE, Mummery CL, Orlova VV (2020). Generation and Functional Characterization of Monocytes and Macrophages Derived from Human Induced Pluripotent Stem Cells. Curr Protoc Stem Cell Biol..

[56] Hickman SE (2013). The microglial sensome revealed by direct RNA sequencing. Nat Neurosci..

[57] Bennett ML (2016). New tools for studying microglia in the mouse and human CNS. Proc. Natl. Acad. Sci..

[58] Galatro TF (2017). Transcriptomic analysis of purified human cortical microglia reveals age-associated changes. Nat. Neurosci..

[59] Gosselin, D. et al. An environment-dependent transcriptional network specifies human microglia identity. *Science* **356**10.1126/science.aal3222 (2017). 10.1126/science.aal3222 PMC5858585 28546318

[60] Ormel PR (2020). A characterization of the molecular phenotype and inflammatory response of schizophrenia patient-derived microglia-like cells. Brain Behav Immun..

[61] Zhang Y (2013). Rapid single-step induction of functional neurons from human pluripotent stem cells. Neuron.

[62] Ho SM (2016). Rapid Ngn2-induction of excitatory neurons from hiPSC-derived neural progenitor cells. Methods.

