# Supplementary material for: Regulation of synaptic connectivity in schizophrenia spectrum by mutual neuron-microglia interaction
Source: Commun Biol. 2023 Apr 29;6:472. doi: 10.1038/s42003-023-04852-9 (PMC10147621; doi:10.1038/s42003-023-04852-9)
Supplement: Supplementary file 1 — Supplementary Information [file 42003_2023_4852_MOESM1_ESM.pdf]

Supplementary Figure 1.

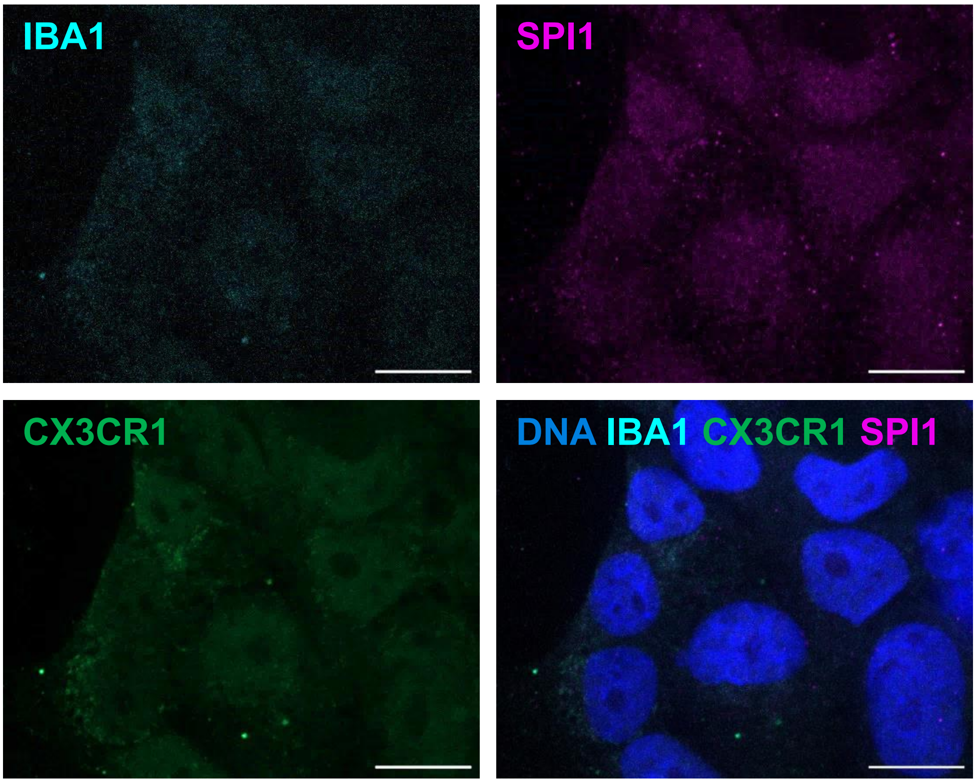

Supplementary Figure 1. Representative immunocytochemical staining of naïve iPSC that do not express microglia-specific markers, such as IBA1, CX3CR1 or SPI1. The brightness of the individual images was adjusted to the maximum to show absence of markers. Scale bar 20 µm.

Supplementary Figure 2.

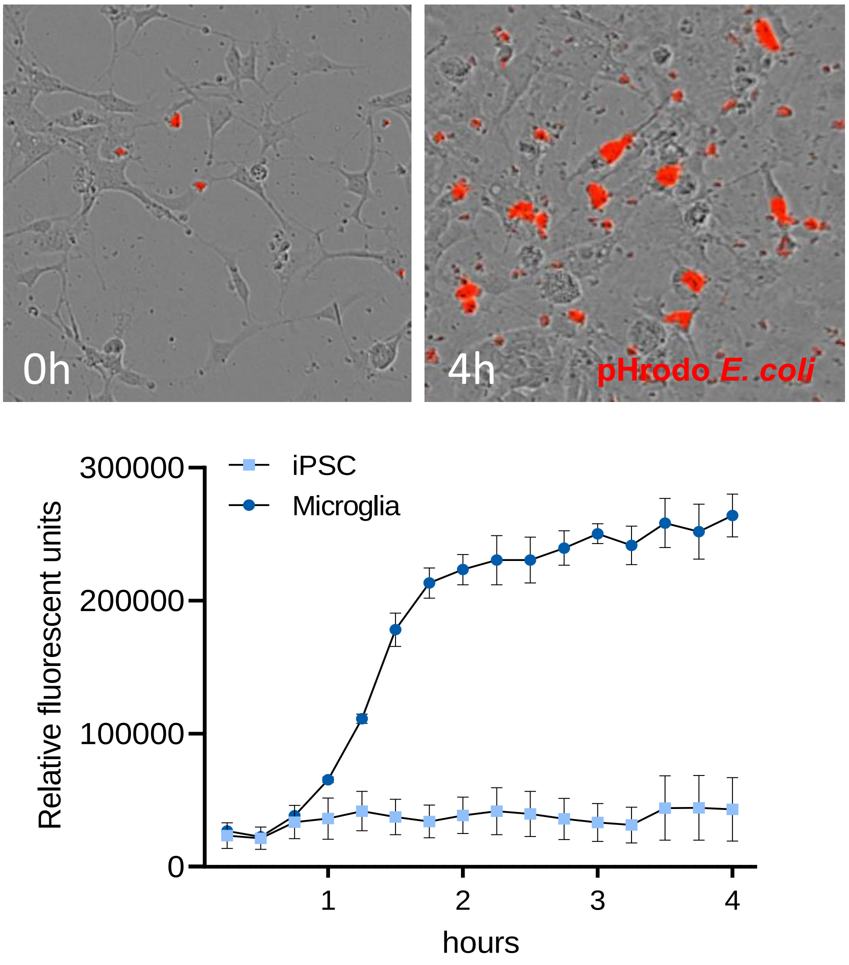

Supplementary Figure 2. iPSC-derived microglia are able to take up *Escherichia coli*-derived bioparticles that are labelled with a pH-sensitive dye (pHrodo Red). Fluorescence increases upon phagocytosis of particles by microglia. Differentiated, day 19 microglia and separately, naive iPSC as negative control, were incubated with *E. coli* bioparticles and the change in fluorescence was measured by live-cell imaging over a period of 4 hours with nine images per well being taken every 15 minutes. Data are represented as mean ± SEM.

Supplementary Figure 3.

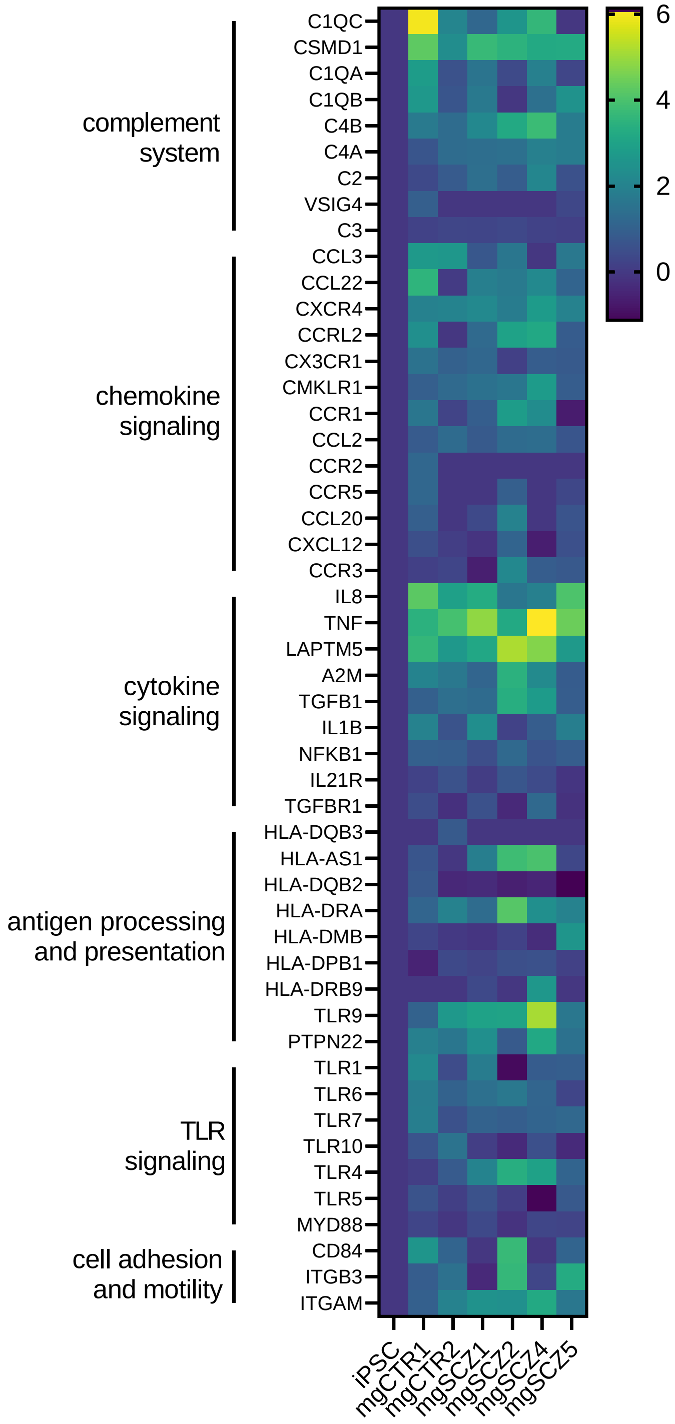

Supplementary Figure 3. iPSC-derived microglia reveal expression of a variety of immune-related genes as revealed by RNA sequencing. Displayed is the log2 fold change (normalized read counts +1) of microglia to naive iPSC. (n = 3 for each group)

Supplementary Figure 4.

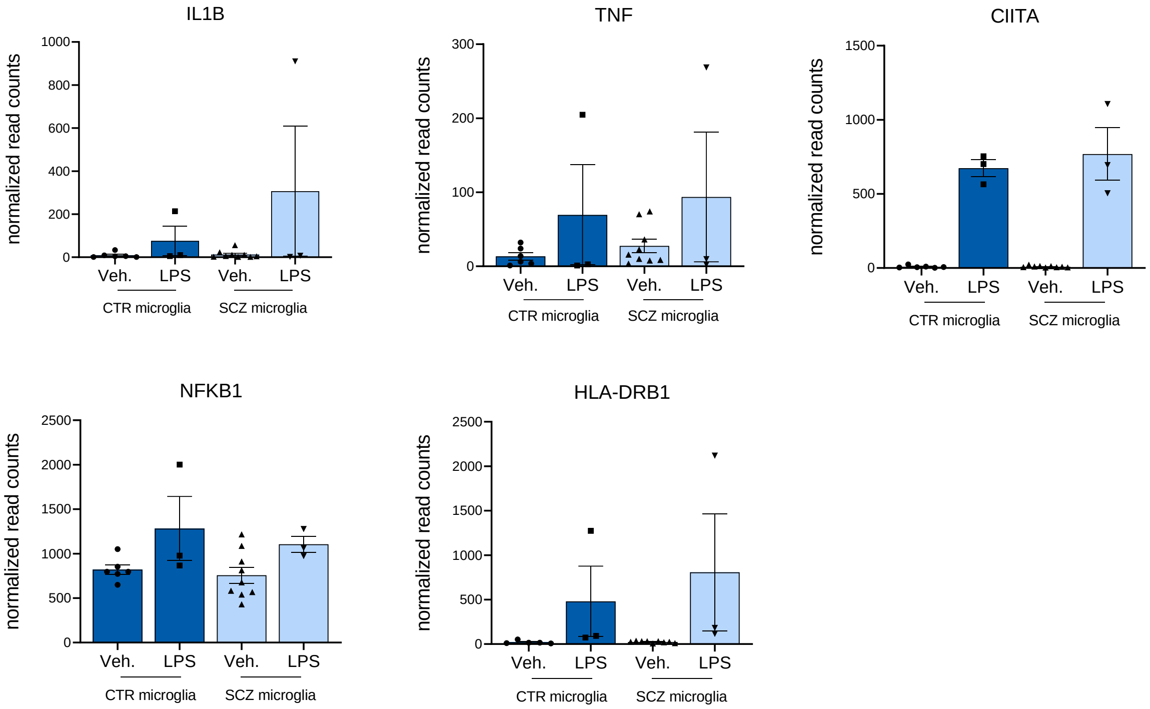

Supplementary Figure 4. RNA sequencing reveals that both microglia from CTR and SCZ donors respond to LPS stimulation with increased expression of pro-inflammatory genes. The number of points represents the number of biological replicates. Data are represented as mean ± SEM. (IL1B: CTR microglia Veh. n = 6; CTR microglia LPS n = 3; SCZ microglia Veh. n = 9; SCZ microglia LPS n = 3; TNF: CTR microglia Veh. n = 6; CTR microglia LPS n = 3; SCZ microglia Veh. n = 9; SCZ microglia LPS n = 3; CIITA: CTR microglia Veh. n = 6; CTR microglia LPS n = 3; SCZ microglia Veh. n = 9; SCZ microglia LPS n = 3; NFKB1: CTR microglia Veh. n = 6; CTR microglia LPS n = 3; SCZ microglia Veh. n = 9; SCZ microglia LPS n = 3; HLA-DRB1: CTR microglia Veh. n = 5; CTR microglia LPS n = 3; SCZ microglia Veh. n = 9; SCZ microglia LPS n = 3)

Supplementary Figure 5.

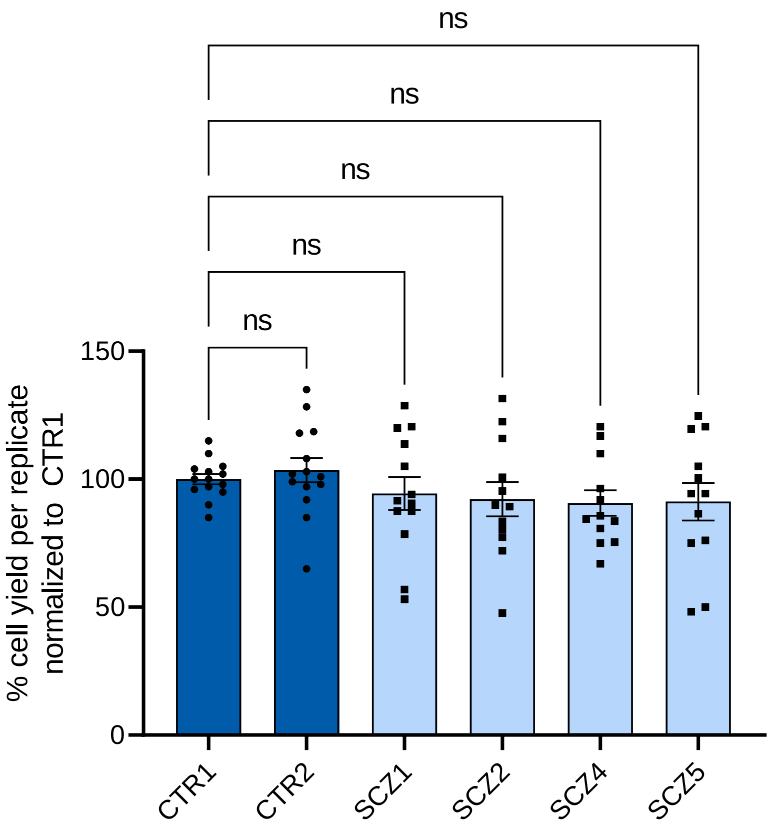

Supplementary Figure 5. Differentiation of CTR and SCZ microglia did not reveal differences in microglia cell yield over several replicates. Each single data point represents the individual cell yield of each line in one biological replicate. Kruskal-Wallis test with Dunn‘s post hoc test, $H(5) = 6.085$, all groups were compared to CTR1. Data are represented as mean ± SEM, (CTR1 n = 12; CTR2 n = 14; SCZ1 n = 13, SCZ2 n = 12; SCZ4 n = 12; SCZ5 n = 12).

Supplementary Figure 6.

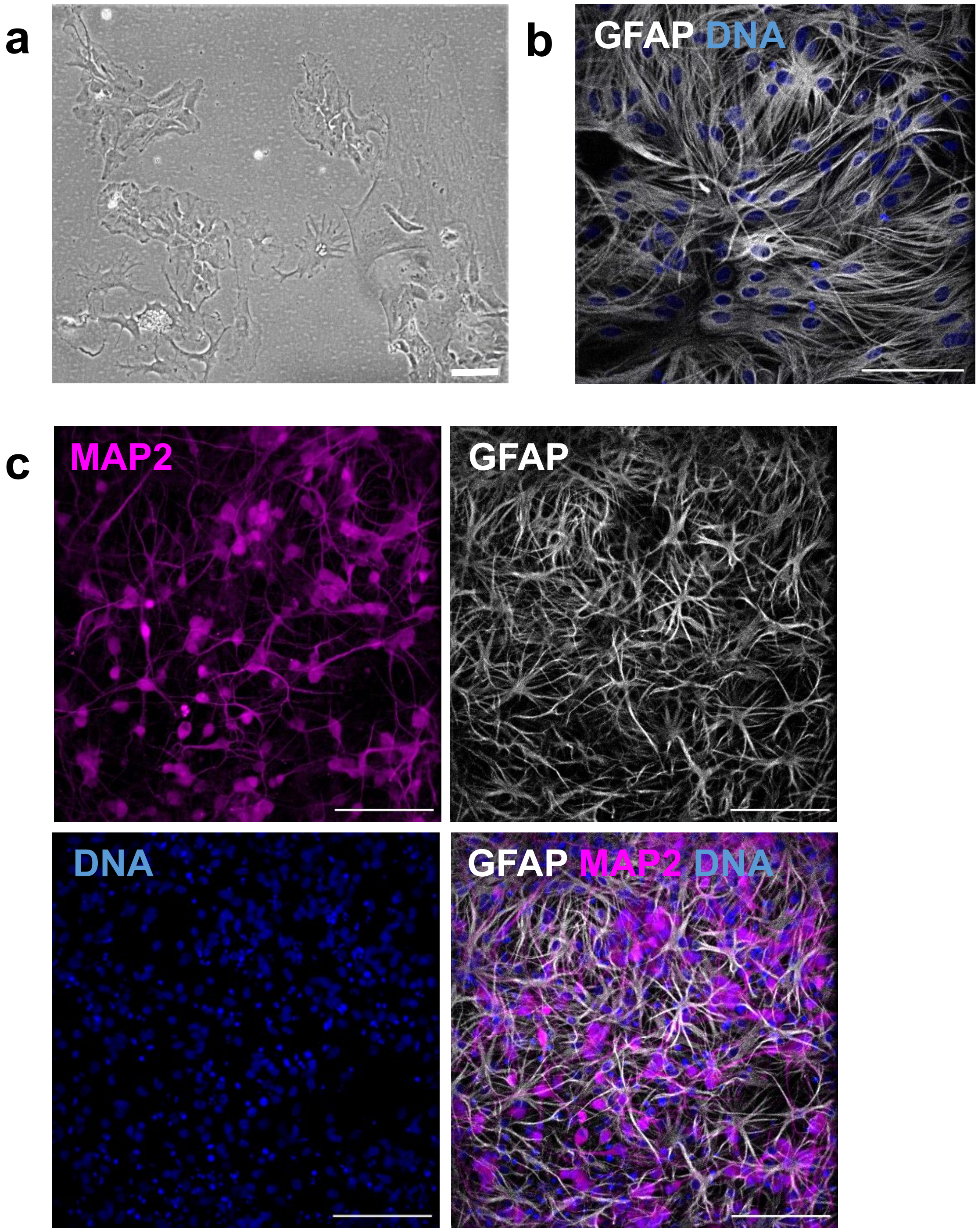

Supplementary Figure 6. Characterization of murine astrocytes that were used routinely in every replicate for improved neuronal culture and maturation. **a** Phase contrast picture of GFAP positive, murine astrocytes 9 days *in vitro* after preparation from newborn mice. Scale bar 100 µm. **b** Fixed and stained astrocytes in mono-culture after 9 days *in vitro*. Scale bar

100 µm. **c** Fixed and stained co-cultures of GFAP positive astrocytes and iPSC-derived MAP2 positive neurons. Scale bar 100 µm.

Supplementary Figure 7.

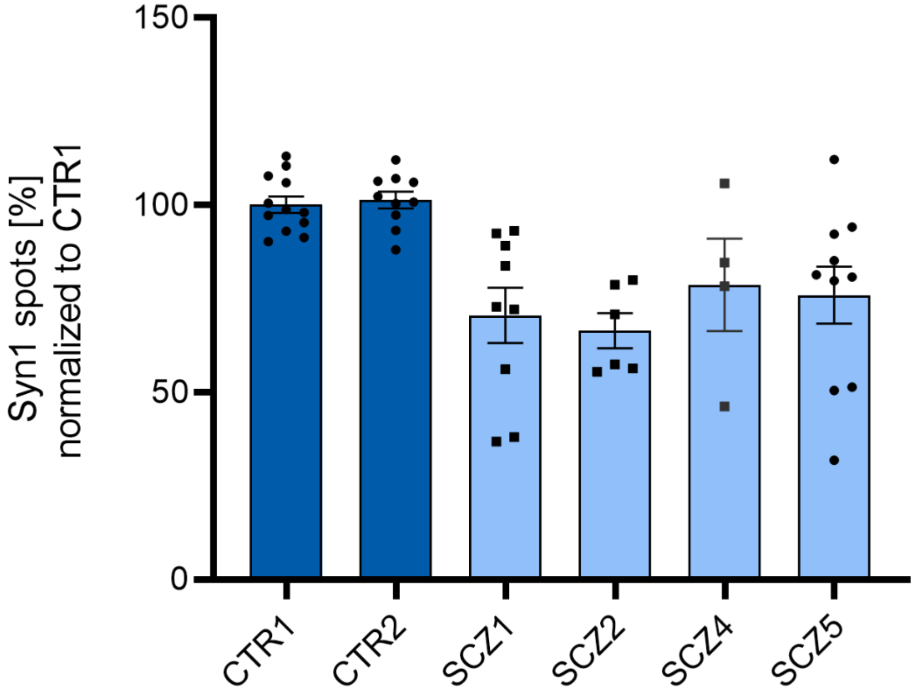

Supplementary Figure 7: Mean SYN1 spot density in individual replicates as representation for the reproducibility and robustness of analyzed parameters. From each clone CTR1 and 2, SCZ 1,2,4 and 5 within one biological replicate (= one independent differentiation) more than four images were analyzed to calculate mean Syn1 spot density represented by a point in the graph. The number of points represents the number of biological replicates. Data are represented as mean ± SEM. (CTR1 n = 12; CTR2 n = 10; SCZ1 n = 9; SCZ2 n = 6; SCZ4 n = 4; SCZ5 n = 10)

Supplementary Figure 8.

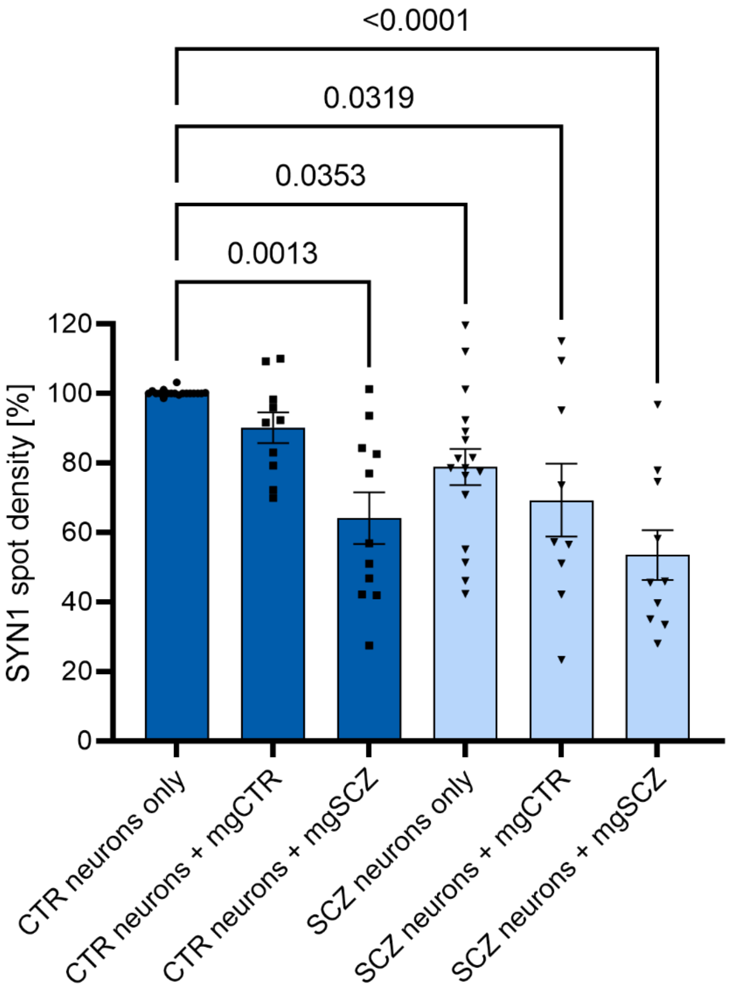

Supplementary Figure 8. Addition of microglia to neuronal cultures leads to a reduction in SYN1 spot density. Presynaptic density as quantified by SYN1 positive spots within a MAP2 positive neuronal network was quantified. Addition of microglia significantly reduces the presynaptic density compared to neuronal cultures without co-cultured microglia. The number of points represents the number of biological replicates. Kruskal-Wallis test with Dunn‘s post hoc test, $H(5) = 30.77$.. Data are normalized to CTR neurons only and are represented as mean ± SEM. (CTR neurons only n = 18; CTR neurons + mgCTR n = 10; CTR neurons + mgSCZ n = 11; SCZ neurons only n = 17; SCZ neurons + mgCTR n = 9; SCZ neurons + mgSCZ n = 10).

Supplementary Figure 9.

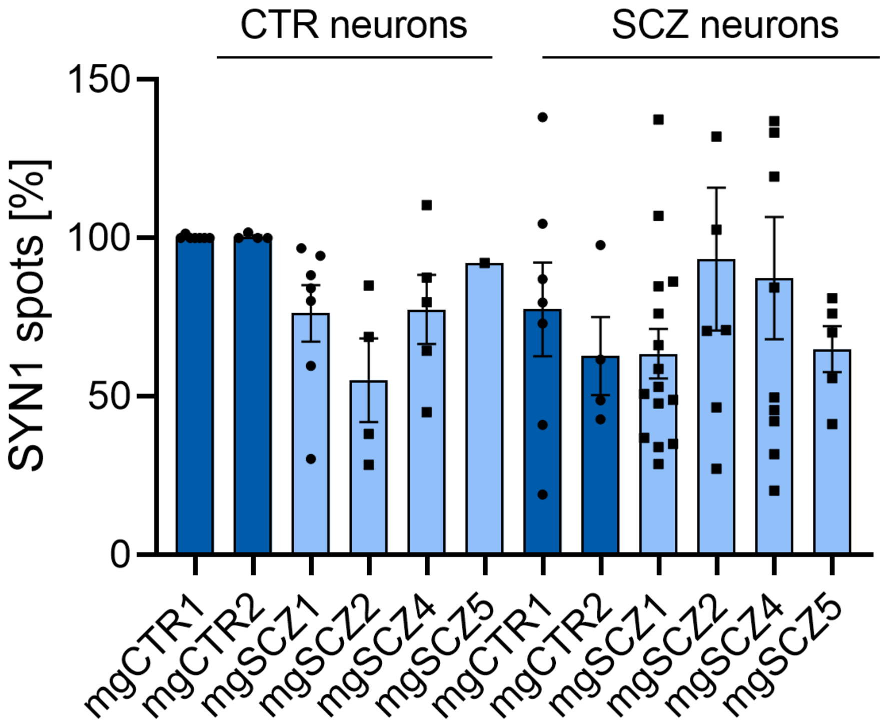

Supplementary Figure 9. Decrease in synaptic density after neuronal co-culture with microglia derived from the individual clones used in this study. Control neurons without added microglia were set 100 %. The number of points represents the number of biological replicates. Data are normalized to CTR neurons only and are represented as mean ± SEM. (CTR neurons: mgCTR1 n = 7, mgCTR2 n = 4, mgSCZ1 n = 7, mgSCZ2 n = 4, mgSCZ3 n = 5, mgSCZ5 n = 1; SCZ neurons: mgCTR n = 7, mgCTR2 n = 4, mgSCZ1 n = 15, mgSCZ2 n = 7, mgCTR1 n = 7, mgCTR2 n = 4, mgSCZ1 n = 15, mgSCZ2 n = 7, mgSCZ4 n = 10, mgSCZ5 n = 5)

Supplementary Figure 10.

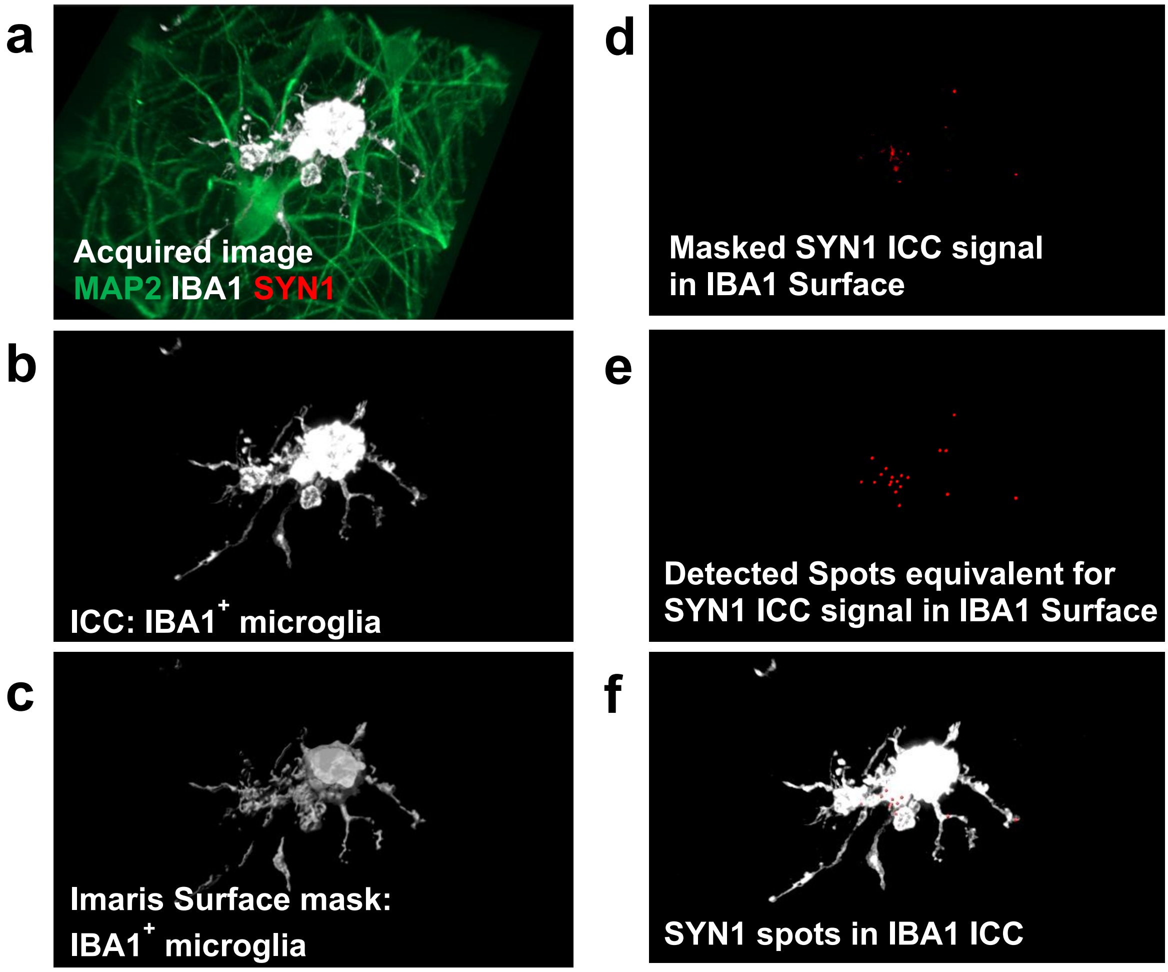

Supplementary Figure 10. 3D Image Analysis using Imaris Bitplane to determine synapse uptake in microglia. **a** Acquired images are saved as 3D z-stacks. **b** The channel for IBA1 positive microglia is selected. **c** A surface mask is created for IBA1 positive microglia with a threshold that covers the whole microglial cell, including areas with lower IBA expression. The threshold is always kept constant throughout the analysis of individual experiments. **d** The created IBA1/microglia surface is masked using the SYN1 channel, leading to a new channel reflecting the SYN1 staining within microglia. **e** Spot detection is used to visualize SYN1 spots within microglia. **f** SYN1 spots are within IBA1 positive microglia.

Supplementary Figure 11.

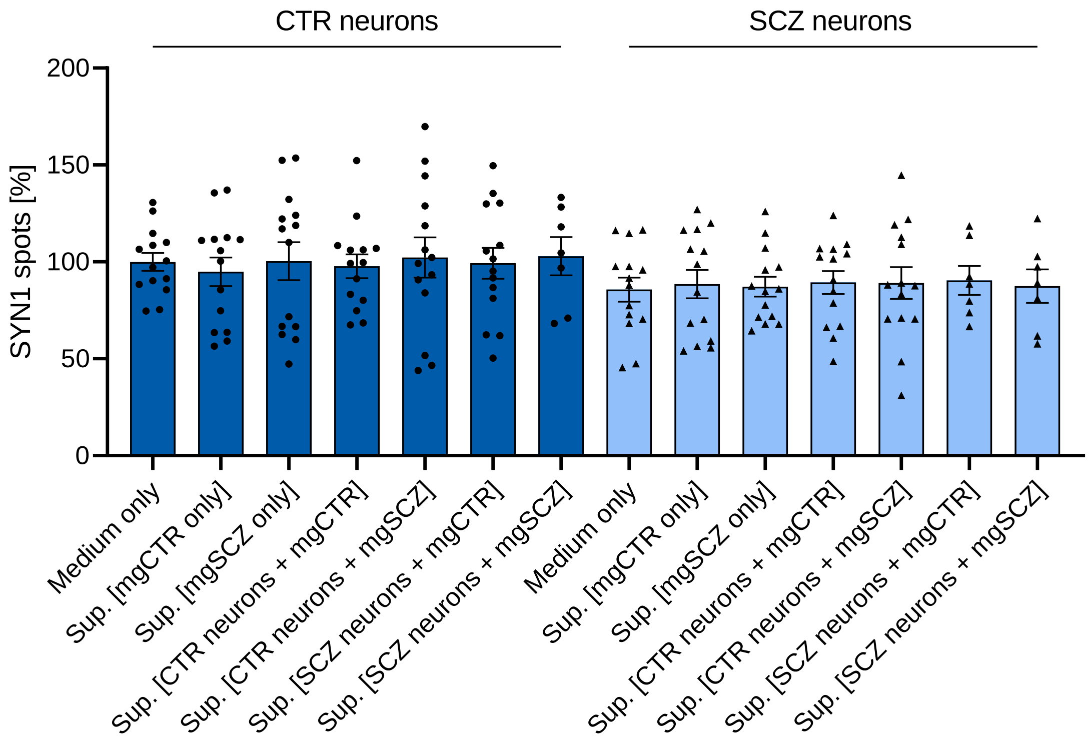

Supplementary Figure 11. CTR and SCZ neurons were incubated with conditioned medium of previous microglia-neuron co-cultures to determine if the direct cell-cell contact is responsible for the observed decline in presynaptic density or if soluble factors induce synapse depletion. CTR neurons and SCZ neurons were cultivated in conditioned medium for 72 hours and SYN1 spots were quantified within MAP2 positive neuronal networks. Co-culture with supernatants led to no significant changes in SYN1 spot density. The number of points represents the number of biological replicates. Data are normalized to CTR neurons in medium only and are represented as mean ± SEM. (CTR neurons: Medium only n = 14; mgCTR only n = 14; mgSCZ only n = 14; CTR neurons + mgCTR n = 14; CTR neurons + mgSCZ n = 14; SCZ neurons + mgCTR n = 14; SCZ neurons + mgSCZ n = 7; SCZ neurons: Medium only n = 14; mgCTR only n = 14; mgSCZ only n = 14; CTR neurons + mgCTR n = 14; CTR neurons + mgSCZ n = 14; SCZ neurons + mgCTR n = 7; SCZ neurons + mgSCZ n = 7)

Supplementary Figure 12.

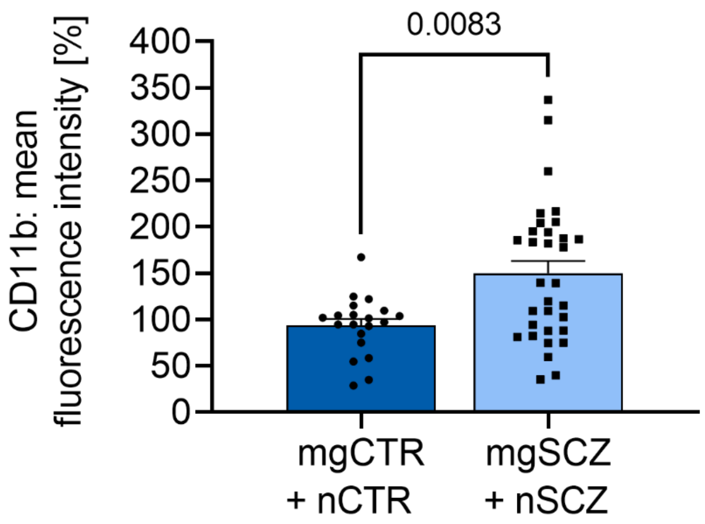

Supplementary Figure 12. CD11b mean fluorescence intensity in co-cultures of [CTR microglia + CTR neurons] and [SCZ microglia + SCZ neurons]. The number of points represents the number of biological replicates. Unpaired, two-tailed Mann Whitney U test was employed for pairwise comparisons, $p = 0.0083$. Data are represented as mean ± SEM. (mgCTR + nCTR n = 20; mgSCZ + nSCZ n = 32).

Supplementary Figure 13.

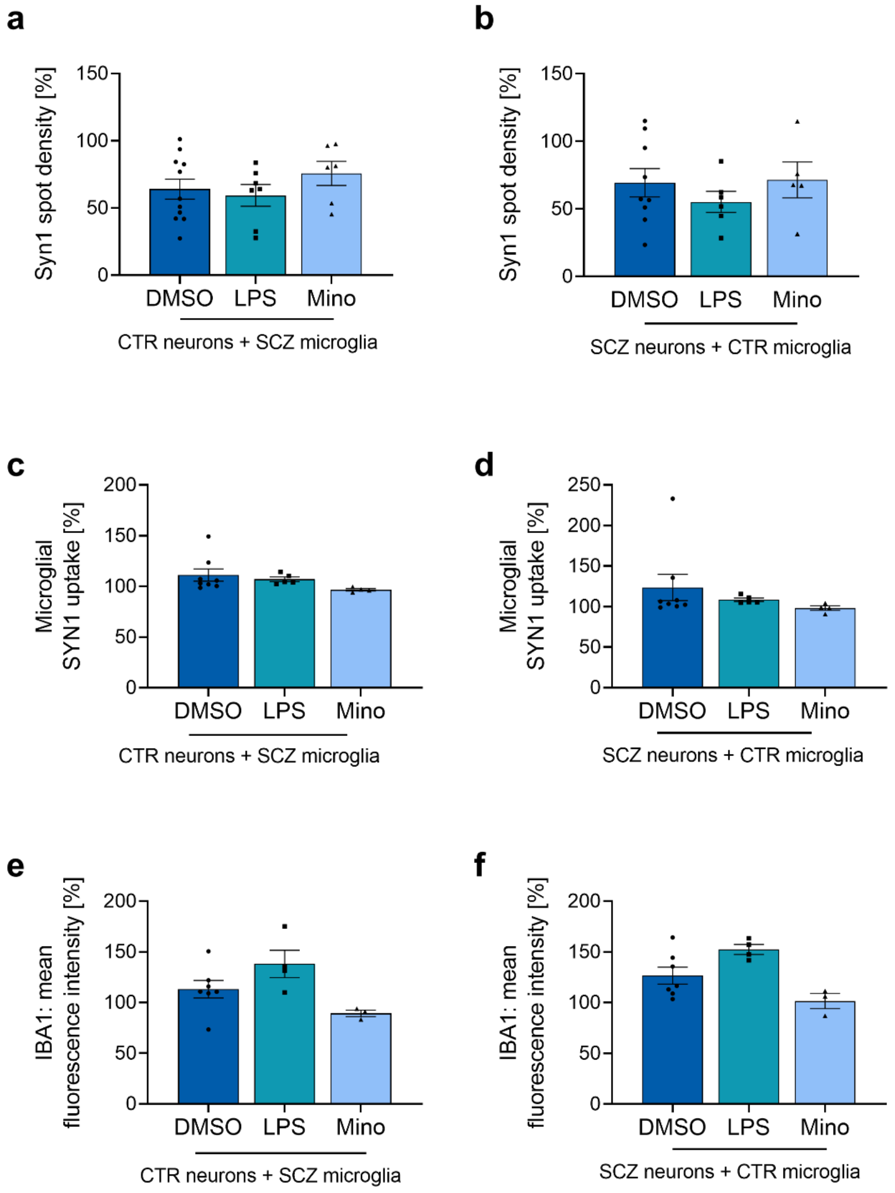

Supplementary Figure 13. SYN1 spots, SYN1 uptake and IBA1 mean fluorescence intensity in cross-combinations of [CTR neurons + SCZ microglia] and [SCZ neurons + CTR microglia]. The number of points represents the number of biological replicates and are represented as mean ± SEM. (**a:** CTR neurons + SCZ microglia: DMSO n = 11, LPS n = 7, Mino n = 6; **b:** SCZ neurons + SCZ microglia: DMSO n = 9, LPS n = 6, Mino n = 5; **c:** CTR neurons + SCZ microglia: DMSO n = 8, LPS n = 5, Mino n = 4; **d:** SCZ neurons + SCZ microglia: DMSO n = 8, LPS n = 5, Mino n = 4; **e:** CTR neurons + SCZ microglia: DMSO n = 7, LPS n = 4, Mino n = 3; **f:** SCZ neurons + SCZ microglia: DMSO n = 7, LPS n = 4, Mino n = 3)

Supplementary Table 1. Selection criteria of SCZ patients.

Inclusion criteria schizophrenia:

- Diagnosis of disease investigated by SCID-I according to DSM-IV
- One first-degree relative also with the diagnosis of a psychotic disorder
- Age: 18 – 65 years

Exclusion criteria:

- Any other diagnosis according to DSM-IV
- No acute episode of psychosis
- Current addiction, except for nicotine and caffeine
- Use of illegal substances within 4 weeks before study
- Mentally handicapped persons
- Pregnancy or lactation
- Suicidal tendency or danger for others

ID	Sex	Age at biopsy	Family history	Diagnosis according to DSM IV	Diagnosis according to DSM5	Medication	Dominant clinical symptoms diagnosed in SCZ patients
SCZ1	male	37	Mother diseased	Schizo-affective disorder / 295.70	Schizo-affective disorder – bipolar type / 295.70	Lithium<br>450 – 0 – 450 mg<br>Quetiapine ret.<br>0 – 0 – 800 mg	cognitive impairment, formal thought disorder, paranoid thoughts
SCZ2	female	54	Sister diseased	Residual type / 295.60	Schizophrenia spectrum / 295.90	Clozapine<br>0 – 100 – 400 mg	formal thought disorder, paranoid thoughts, hallucinations
SCZ4	male	50	Mother + brother diseased	Paranoid type / 295.30	Schizophrenia spectrum / 295.90	Amisulpride<br>150 mg – 0 – 0<br>Mirtazapine<br>0 – 0 – 0 – 30 mg	formal thought disorder, paranoid thoughts, persecutional delusion
SCZ5	female	27	Mother diseased	Schizo-affective disorder / 295.70	Schizo-affective disorder – bipolar type / 295.70	Olanzapine<br>0 – 0 – 7,5 mg<br>Quetiapine<br>50 mg if required	paranoid thoughts, persecutional delusion, mania, mutism

Supplementary Table 2. Significantly deregulated genes in untreated CTR microglia vs. untreated SCZ microglia.

Gene ID	Gene	Log2 Fold Change	P-value	FDR P-value
ENSG00000053438	NNAT	-8,80	2,16E-39	1,06E-34
ENSG00000134184	GSTM1	-7,80	1,14E-13	2,81E-09
ENSG00000022556	NLRP2	-7,60	2,66E-12	4,35E-08
ENSG00000231007	CDC20P1	-4,33	3,80E-11	4,67E-07
ENSG00000197582	GPX1P1	-5,26	9,28E-11	9,13E-07
ENSG00000152726	FAM21B	-1,78	2,35E-09	1,93E-05
ENSG00000267374	LINC00669	-3,48	2,48E-08	1,74E-04
ENSG00000267270	AC139100.2	-2,37	6,02E-07	3,70E-03
ENSG00000013297	CLDN11	-2,55	7,01E-07	3,83E-03
ENSG00000262074	SNORD3B-2	-2,17	7,96E-07	3,91E-03
ENSG00000230567	FAM203B	-6,61	1,44E-06	6,45E-03
ENSG00000204169	AGAP7	-4,58	2,57E-06	1,05E-02
ENSG00000198558	HIST1H4L	2,88	2,78E-06	1,05E-02
ENSG00000135828	RNASEL	-2,03	6,12E-06	2,15E-02
ENSG00000240666	MME-AS1	-5,41	7,42E-06	2,39E-02
ENSG00000183506	PI4KAP2	-0,87	7,78E-06	2,39E-02
ENSG00000168497	SDPR	-2,82	1,00E-05	2,90E-02
ENSG00000236816	ANKRD20A7P	-3,24	1,14E-05	3,12E-02

Differentially expressed genes were identified at a significance threshold of $p < 0.05$ (FDR corrected p-values). iPSC were derived from two controls ($n = 2$) and microglia were derived from four patients and two healthy controls in three independent experiments ($n = 18$).
