# Supplementary material for: Regulation of synaptic connectivity in schizophrenia spectrum by mutual neuron-microglia interaction
Source: Commun Biol. 2023 Apr 29;6:472. doi: 10.1038/s42003-023-04852-9 (PMC10147621; doi:10.1038/s42003-023-04852-9)
Supplement: Supplementary file 2 — Description of Additional Supplementary Files [file 42003_2023_4852_MOESM2_ESM.docx]

**Description of Additional Supplementary Files**

**File name:** Supplementary Data 1

**Description:** The source data behind the manuscript
