# Supplementary material for: Regulation of synaptic connectivity in schizophrenia spectrum by mutual neuron-microglia interaction
Source: Commun Biol. 2023 Apr 29;6:472. doi: 10.1038/s42003-023-04852-9 (PMC10147621; doi:10.1038/s42003-023-04852-9)
Supplement: Supplementary file 4 — Reporting Summary [file 42003_2023_4852_MOESM4_ESM.pdf]

nature portfolio

Corresponding author(s): Hansjürgen Volkmer

Last updated by author(s): Mar 31, 2023

Reporting Summary

Nature Portfolio wishes to improve the reproducibility of the work that we publish. This form provides structure for consistency and transparency in reporting. For further information on Nature Portfolio policies, see our Editorial Policies and the Editorial Policy Checklist.

Statistics

For all statistical analyses, confirm that the following items are present in the figure legend, table legend, main text, or Methods section.

n/a	Confirmed	
☐	☒	The exact sample size (*n*) for each experimental group/condition, given as a discrete number and unit of measurement
☐	☒	A statement on whether measurements were taken from distinct samples or whether the same sample was measured repeatedly
☐	☒	The statistical test(s) used AND whether they are one- or two-sided<br>*Only common tests should be described solely by name; describe more complex techniques in the Methods section.*
☐	☒	A description of all covariates tested
☐	☒	A description of any assumptions or corrections, such as tests of normality and adjustment for multiple comparisons
☐	☒	A full description of the statistical parameters including central tendency (e.g. means) or other basic estimates (e.g. regression coefficient) AND variation (e.g. standard deviation) or associated estimates of uncertainty (e.g. confidence intervals)
☐	☒	For null hypothesis testing, the test statistic (e.g. *F*, *t*, *r*) with confidence intervals, effect sizes, degrees of freedom and *P* value noted<br>*Give P values as exact values whenever suitable.*
☒	☐	For Bayesian analysis, information on the choice of priors and Markov chain Monte Carlo settings
☐	☒	For hierarchical and complex designs, identification of the appropriate level for tests and full reporting of outcomes
☒	☐	Estimates of effect sizes (e.g. Cohen's *d*, Pearson's *r*), indicating how they were calculated

Our web collection on statistics for biologists contains articles on many of the points above.

Software and code

Policy information about availability of computer code

Data collection	no custom code was used
Data analysis	no custom code was used

For manuscripts utilizing custom algorithms or software that are central to the research but not yet described in published literature, software must be made available to editors and reviewers. We strongly encourage code deposition in a community repository (e.g. GitHub). See the Nature Portfolio guidelines for submitting code & software for further information.

Data

Policy information about availability of data

All manuscripts must include a data availability statement. This statement should provide the following information, where applicable:

- Accession codes, unique identifiers, or web links for publicly available datasets
- A description of any restrictions on data availability
- For clinical datasets or third party data, please ensure that the statement adheres to our policy

Cell line unique identifiers of all iPS cell lines are: NMIi001-A, NMIi010-A, NMIi002-A, NMIi004-A, NMIi005-A, NMIi006-A. All cell lines used in this study are registered at the Human pluripotent stem cell registry (hpscreg.eu). RNA sequencing data are available for download from the NCBI Gene Expression Omnibus (GEO) (NCBI GEO no. GSE213232: https://www.ncbi.nlm.nih.gov/geo/query/acc.cgi?acc=GSE213232). Additional data that support the findings of this study are available from the corresponding authors upon request.

Human research participants

Policy information about studies involving human research participants and Sex and Gender in Research.

Reporting on sex and gender	Biological samples were taken from male and female individuals (sex). The impact of sex was not studied in the experiments. Groups were formed according to healthy control and schizophrenia spectrum. Gender data were not collected.
Population characteristics	Population characteristics are depicted in supplementary table 2
Recruitment	Recruitment was performed by a MD in the Psychiatry Dept. Univ. of Tübingen. Inclusion and exclusion criteria are summerized in supplementary table 2
Ethics oversight	The methods were performed in accordance with relevant guidelines and regulations and approved by the Ethics Committee of the University Hospital and Faculty of Medicine Tuebingen (Approval number: 311/2013BO1).

Note that full information on the approval of the study protocol must also be provided in the manuscript.

Field-specific reporting

Please select the one below that is the best fit for your research. If you are not sure, read the appropriate sections before making your selection.

☒ Life sciences ☐ Behavioural & social sciences ☐ Ecological, evolutionary & environmental sciences

For a reference copy of the document with all sections, see nature.com/documents/nr-reporting-summary-flat.pdf

Life sciences study design

All studies must disclose on these points even when the disclosure is negative.

Sample size	No statistical calculation was performed to pre-determine sample sizes. The sample size was determined based on our previous experience and standards in the field. A minimum of three independent biological replicates was performed for each experiment as indicated in figure legends of individual experiments. We believe this to be sufficient since all experiments were highly reproducible within these replicates.
Data exclusions	For analyses using confocal microscopy, images were taken of neuronal networks with a comparable density and a comparable size of the acquired Z-stack. Images with low neuronal network density were excluded from the analysis. No differences in number of such images were noted between schizophrenia subjects and healthy controls.
Replication	Experimental findings were reproduced successfully over at least 3 independent differentiations as indicated in figure legends of individual experiments.
Randomization	Patient or control-derived iPS cells were not allocated randomly into groups. Patients were selected for a family history with schizophrenia to secure a strong genetic component.
Blinding	Investigators were blinded to group allocation during data analysis.

Reporting for specific materials, systems and methods

We require information from authors about some types of materials, experimental systems and methods used in many studies. Here, indicate whether each material, system or method listed is relevant to your study. If you are not sure if a list item applies to your research, read the appropriate section before selecting a response.

Materials & experimental systems

n/a	Involved in the study	
☐	☒	Antibodies
☐	☒	Eukaryotic cell lines
☒	☐	Palaeontology and archaeology
☐	☒	Animals and other organisms
☒	☐	Clinical data
☒	☐	Dual use research of concern

Methods

n/a	Involved in the study	
☒	☐	ChIP-seq
☐	☒	Flow cytometry
☒	☐	MRI-based neuroimaging

Antibodies

Antibodies used	1.) mouse monoclonal anti-Beta-Tubulin III (STEMCELL Technologies, clone AA10, catalog no. 60100, 1:250)<br>2.) mouse monoclonal CX3CR1 (BioLegend, clone K0124E1, catalog no. 355701, 1:500)<br>3.) rabbit polyclonal anti-IBA1 (FUJIFILM Wako Chemicals, catalog no. 019-19741, 1:1000)<br>4.) rat monoclonal anti-LAMP1 (Santa Cruz Biotechnology, clone 1D4B, catalog no. sc-19992, 1:100)<br>5.) chicken polyclonal anti-MAP2 (Invitrogen, catalog no. PA1-10005; 1:2500)<br>6.) mouse monoclonal anti-NFκB p65 (Cell Signaling, clone L8F6, catalog no. 6956)<br>7.) mouse monoclonal anti-PAX6 (BioLegend, clone AD2.35, catalog no. 862001, 1:200)<br>8.) Phalloidin CruzFluor# 488 (Santa Cruz Biotechnology, catalog no. sc-363791)<br>9.) rabbit monoclonal recombinant anti-PSD95 (Synaptic Systems, catalog no. 124008, 1:500)<br>10.) mouse monoclonal anti-SPI1 (PU.1, BioLegend, clone 7C6B05, catalog no. 658002, 1:100)<br>11.) rabbit polyclonal anti-SOX1 (Abcam, catalog no. ab22572, 1:500)<br>12.) mouse monoclonal anti-Synapsin1 (Synaptic Systems, clone 46.1, catalog no. 106011, 1:1000)<br>13.) rabbit polyclonal anti-Synaptophysin1 (Synaptic Systems, catalog no. 101002, 1:500)<br>14.) rabbit polyclonal anti-TMEM119 (Synaptic Systems, catalog no. 400002, 1:400)<br>15.) rabbit monoclonal anti-TREM2 (Cell Signaling, clone D8I4C, catalog no. 91068, 1:400)<br>16.) mouse monoclonal anti-VGlut1 (Synaptic Systems, clone 317G6, catalog no. 135511, 1:300)<br>17.) mouse monoclonal anti-Nestin (Synaptic System, clone JP63, catalog no. 312011, 1:1000)<br>18.) anti-human SSEA-4 PE-Vio770 (Miltenyi Biotech, clone REA101, catalog no. 130-105-081)<br>19.) anti-human CD11b FITC (Thermo Fisher Scientific, clone ICRF44, catalog no. 11-0118-42)<br>20.) anti-human CD45 VioBlue (Miltenyi Biotech, clone REA747, catalog no. 130-110-775)<br>21.) rabbit monoclonal recombinant anti-PSD95 (Synaptic Systems, catalog no. 124008, 1:500)
Validation	In-house validation was performed by secondary antibody only staining controls. Below are citations from the individual manfacturer's homepages.<br>1.) anti-Beta-Tubulin III (STEMCELL Technologies, clone AA10, catalog no. 60100, 1:250) is verified for ICC, IF, WB: "This antibody clone has been verified for labeling neural stem and progenitor cells..."<br>2.) CX3CR1 (BioLegend, clone K0124E1, catalog no. 355701, 1:500):<br>3.) IBA1 (FUJIFILM Wako Chemicals, catalog no. 019-19741, 1:1000): "Specific to microglia and macrophage, but not cross-reactive with neuron and astrocyte."<br>4.) LAMP1 (Santa Cruz Biotechnology, clone 1D4B, catalog no. sc-19992, 1:100)<br>5.) MAP2 (Invitrogen, catalog no. PA1-10005; 1:2500): "This Antibody was verified by Relative expression to ensure that the antibody binds to the antigen stated."<br>6.) NFκB p65 (Cell Signaling, clone L8F6, catalog no. 6956): "This antibody has been validated using SimpleChIP® Enzymatic Chromatin IP Kits. NF-κB p65 (L8F6) Mouse mAb recognizes endogenous levels of total NF-κB p65 protein."<br>7.) mouse monoclonal anti-PAX6 (BioLegend, clone AD2.35, catalog no. 862001, 1:200): "verified for IHC-P, IHC-F, WB and quality tested for ICC. Each lot of this antibody is quality control tested by immunocytochemistry."<br>8.) Phalloidin CruzFluor# 488 (Santa Cruz Biotechnology, catalog no. sc-363791)<br>9.) SPI1 (PU.1, BioLegend, clone 7C6B05, catalog no. 658002, 1:100): "verified for immunocytochemistry"<br>10.) SOX1 (Abcam, catalog no. ab22572, 1:500): "Our Abpromise guarantee covers the use of ab22572 in the following tested applications: ICC/IF"<br>11.) Synapsin1 (Synaptic Systems, clone 46.1, catalog no. 106011, 1:1000): "Specific for synapsin 1a and 1b independent of phosphorylaion state, K.O. verified".<br>12.) Synaptophysin1 (Synaptic Systems, catalog no. 101002, 1:500): "Specific for synaptophysin 1, no cross-reactivity to other synaptophysins".<br>13.) TMEM119 (Synaptic Systems, catalog no. 400002, 1:400) is "specific for TMEM 119".<br>14.) TREM2 (Cell Signaling, clone D8I4C, catalog no. 91068, 1:400) "recognizes endogenous levels of total TREM2 protein."<br>15.) VGlut1 (Synaptic Systems, clone 317G6, catalog no. 135511, 1:300) is " specific for VGLUT1, K.O. verified<br>16.) Nestin (Synaptic System, clone JP63, catalog no. 312011, 1:1000) is "specific for human nestin".<br>17.) SSEA-4 PE-Vio770 (Miltenyi Biotech, clone REA101, catalog no. 130-105-081): "Extended validation. In order to compare the epitope specificity of an antibody, the clone being used is compared with other known clones recognizing the same antigen in a competition assay."<br>18.) CD11b FITC (Thermo Fisher Scientific, clone ICRF44, catalog no. 11-0118-42): "This IRCF44 antibody has been pre-titrated and tested by flow cytometric analysis of normal human peripheral blood cells."<br>19.) CD45 VioBlue (Miltenyi Biotech, clone REA747, catalog no. 130-110-775): "Extended validation. In order to compare the epitope specificity of an antibody, the clone being used is compared with other known clones recognizing the same antigen in a competition assay."<br>20.) rabbit monoclonal recombinant anti-PSD95 (Synaptic Systems, catalog no. 124008) "validated, Reacts with: rat (P31016), mouse (Q62108), chicken, human (P78352)"

Eukaryotic cell lines

Policy information about cell lines and Sex and Gender in Research

Cell line source(s)	Skin biopsies from schizophrenia patients and one healthy volunteer and peripheral blood for CD34+ progenitor cell isolation from one healthy volunteer
Authentication	Short tandem repeat analysis
Mycoplasma contamination	all cell lines used in this study were regularly tested negative for mycoplasma contamination

Commonly misidentified lines (See ICLAC register)	no commonly misidentified lines were used in this study

Animals and other research organisms

Policy information about studies involving animals; ARRIVE guidelines recommended for reporting animal research, and Sex and Gender in Research

Laboratory animals	for primary astrocyte preparation, female and pregnant RjOrl:SWISS wild type mice were used in this study
Wild animals	no wild animals were used in this study
Reporting on sex	astrocytes from embryos were prepared and collected in one batch irrespective of sex.
Field-collected samples	this study does not involve samples collected from the field
Ethics oversight	the study was approved by the regional authority in Baden-Wuerttemberg, Tuebingen

Note that full information on the approval of the study protocol must also be provided in the manuscript.

Flow Cytometry

Plots

Confirm that:

- [x] The axis labels state the marker and fluorochrome used (e.g. CD4-FITC).
- [x] The axis scales are clearly visible. Include numbers along axes only for bottom left plot of group (a 'group' is an analysis of identical markers).
- [ ] All plots are contour plots with outliers or pseudocolor plots.
- [x] A numerical value for number of cells or percentage (with statistics) is provided.

Methodology

Sample preparation	Day 0 iPSC, day 10 and day 19 microglia of the differentiation were used for flow cytometric analysis of surface markers. Live cells were enzymatically detached using accutase and stained using three surface markers (SSEA-4 PE-Vio770, CD11b FITC and CD45 VioBlue) for 30 Min at 4°C. Subsequently, cells were washed three times using PBS + 1% FCS and lastly, resuspended in 500 µl PBS. Flow cytometric analysis followed immediately.
Instrument	BD FACSMelody from BD Biosciences
Software	For data collection, software FACS Chorus (BD Biosciences) was used. For data analysis, software FlowJo (v.10-6-1, FlowJo LLC) was used.
Cell population abundance	n/a
Gating strategy	Doublets were excluded in FSC and SSC. Unstained cells served as negative population.

- [ ] Tick this box to confirm that a figure exemplifying the gating strategy is provided in the Supplementary Information.
